# Splicing factors control triple-negative breast cancer cell mitosis through SUN2 interaction and sororin intron retention

**DOI:** 10.1186/s13046-021-01863-4

**Published:** 2021-03-01

**Authors:** Esmee Koedoot, Eline van Steijn, Marjolein Vermeer, Román González-Prieto, Alfred C. O. Vertegaal, John W. M. Martens, Sylvia E. Le Dévédec, Bob van de Water

**Affiliations:** 1 Division of Drug Discovery and Safety, LACDR, Leiden University, Einsteinweg 55, 2333 CC Leiden, The Netherlands; 2 Department of Molecular Cell Biology, Leiden University Medical Center, Leiden, The Netherlands; 3 Department of Medical Oncology and Cancer Genomics Netherlands, Erasmus MC Cancer Institute, Erasmus University Medical Center, Rotterdam, the Netherlands

**Keywords:** Triple-negative breast cancer, Mitosis, Sister chromatid cohesion, RNA splicing, Splicing factors, Proliferation

## Abstract

**Background:**

Triple negative breast cancer (TNBC) is an aggressive subtype of breast cancer with limited therapeutic opportunities. Recently, splicing factors have gained attention as potential targets for cancer treatment. Here we systematically evaluated the role of RNA splicing factors in TNBC cell proliferation.

**Methods:**

In this study, we performed an RNAi screen targeting 244 individual splicing factors to systematically evaluate their role in TNBC cell proliferation. For top candidates, mechanistic insight was gained using amongst others western blot, PCR, FACS, molecular imaging and cloning. Pulldown followed by mass spectrometry were used to determine protein-protein interactions and patient-derived RNA sequencing data was used relate splicing factor expression levels to proliferation markers.

**Results:**

We identified nine splicing factors, including SNRPD2, SNRPD3 and NHP2L1, of which depletion inhibited proliferation in two TNBC cell lines by deregulation of sister chromatid cohesion (SCC) via increased sororin intron 1 retention and down-regulation of SMC1, MAU2 and ESPL1. Protein-protein interaction analysis of SNRPD2, SNRPD3 and NHP2L1 identified that seven out of the nine identified splicing factors belong to the same spliceosome complex including novel component SUN2 that was also critical for efficient sororin splicing. Finally, sororin transcript levels are highly correlated to various proliferation markers in BC patients.

**Conclusion:**

We systematically determined splicing factors that control proliferation of breast cancer cells through a mechanism that involves effective sororin splicing and thereby appropriate sister chromatid cohesion. Moreover, we identified SUN2 as an important new spliceosome complex interacting protein that is critical in this process. We anticipate that deregulating sororin levels through targeting of the relevant splicing factors might be a potential strategy to treat TNBC.

**Supplementary Information:**

The online version contains supplementary material available at 10.1186/s13046-021-01863-4.

## Background

Breast cancer is the most prevalent cancer in women. Triple-negative breast cancer (TNBC) is a breast cancer subtype lacking expression of the estrogen receptor (ER), progesterone receptor (PR) and human epidermal growth factor receptor 2 (HER2) [1]. Although TNBC only accounts for 15% of all breast cancer cases [2], it is disproportionately responsible for breast cancer related deaths compared to other breast cancer subtypes [3]. In contrast to ER positive breast cancer, no targeted therapy is yet available for TNBC and therefore the current treatment strategy still consists of surgery and unselective aggressive treatments, such as chemo- and radiotherapy [4]. Therefore, unraveling the underlying mechanisms of TNBC as well as development of effective targeted therapies are essential to reduce the mortality caused by this disease.

Accumulating evidence suggests that RNA splicing is critical for (breast) cancer progression [5–8]. Splicing is a post-transcriptional process essential for pre-mRNA maturation in which non-coding regions (introns) are removed and coding regions (exons) are ligated. Moreover, splicing creates an extra layer of gene control; selective inclusion of specific exons or introns into the final transcript can result in functionally different isoforms from the same gene, also called alternative splicing. Many alternative splicing events have been linked to different pathways important in cancer development and progression, such as apoptosis (Bcl-x, caspase 2, Fas), metabolism (pyruvate kinase M), oncogenes (Ron, Rac1, FGFRs, CD44, BRAF), tumor suppressor genes (p53) and angiogenesis (VEGF) [5, 8]. The splicing procedure is a complex multistep process catalyzed by the spliceosome; a highly dynamic complex consisting of five small nuclear ribonucleoproteins (snRNPs) and many associated proteins. In total, there are approximately 250 proteins involved in catalyzing the splicing reaction (also called splicing factors), each playing a specific role during the splicing process [9]. Recently, some studies reported a link between splicing factor deficiency, defective splicing and aberrant chromosome segregation during mitosis, mediated by a premature loss of sister chromatid cohesion (SCC) [10, 11]. Also, some splicing factors such as PRPF4B and SF3B3 have been linked to hormone receptor signaling in breast cancer proliferation [12, 13]. Altogether these studies suggest a relation between splicing, mitosis and cancer growth, providing a potential strategy to combat cancer. Since the role of splicing factors in TNBC has not yet been evaluated, we performed an RNAi screen to systematically evaluate the role of the 244 splicing factors in TNBC proliferation. We discovered nine splicing factors (AQR, CRNKL1, MFAP1, NHP2L1, PRPF8, SF3B1, SNRPD2, SNRPD3 and SNRPF), of which knockdown significantly impaired cell proliferation. Depletion of these factors resulted in disruption of sister chromatid cohesion (SCC) via sororin intron 1 retention and decreased levels of ESPL1, MAU2 and SMC1, leading to a stall in G1-S transition and ultimately cell death. Seven of these nine splicing factors seem to reside in a same complex suggesting that the interaction between these splicing partners is essential for TNBC cell growth. Interestingly, splicing factors SNRPD2, SNRPD3 and NHP2L1 were also interacting with known mitotic proteins such as SUN2, providing another mechanism for the mitotic dependency on RNA splicing. Splicing inhibition by Pladienolide B treatment resulted in impaired TNBC cell proliferation through dysregulated SCC similar to splicing factor knockdown. In conclusion, targeting our identified splicing factors in particular seems to provide a new window of anti-cancer targeted strategy in TNBC.

## Methods

### Cell culture

Hs578T (ATCC-HBT-126), MDA-MB-231 (ATCC-HBT-26), HCC1806 (ATCC-CRL-2335), MDA-MB-468 (ATCC-HTB-132), MCF7 (ATCC-HTB-22) and T47D (ATCC-HTB-133) were purchased from ATCC. Hs578T, MDA-MB-231, HCC1806, MDA-MB-468 and MCF7 were grown in RPMI-1640 medium (Gibco, ThermoFisher Scientific, Breda, The Netherlands). T47D was grown in Dulbecco’s modified Eagle’s medium (DMEM, Gibco, ThermoFisher Scientific, Breda). Both media were supplemented with 10% FBS (GE Healthcare, Landsmeer, The Netherlands), 25 IU/ml penicillin and 25 μg/ml streptomycin (ThermoFisher Scientific) and cells were cultured at 37 °C in a humidified 5% CO2 incubator.

### Antibodies and reagents

Mouse anti-Cyclin B1 (#4135), rabbit anti-CDK9 (#2316), rabbit anti-Aurora A (#3092), rabbit anti-phospho-pRb (#9307), mouse anti-pRb (#2692S), rabbit anti-MCM2 (#3619) and rabbit anti-pHistone-H3 (#9701) were purchased from Cell Signaling. Mouse anti-CDC7 (ab10535), rabbit antiphospho-MCM2 (ab70371), rabbit anti-SNRPD2 (ab155030), rabbit anti-SNRPD3 (ab111094) and rabbit anti-NHP2L1 (ab95958) were obtained from Abcam. Mouse anti-Cyclin D1 (no. sc20044) was purchased from Santa Cruz Biotechnology. Mouse anti-GFP (#11814460001) was purchased from Roche. Rat anti-phospho-RNA polymerase II (no. 04–1571) was from Merck Millipore. Mouse anti-tubulin (no. T-9026) was purchased from Sigma-Aldrich. HRP, Alexa-488 and Alexa-647 tagged secondary antibodies were purchased from Jackson Immunoresearch Laboratories.

### Generation of GFP-tagged cell lines

Human SNRPD2, SNRPD3 and NHP2L1 BAC clones were selected and GFP-tagged as previously described [14, 15] and stably introduced in Hs578T cells using 50 μg/ml G-418 for selection and FACS sorted twice for GFP expression.

### Transient siRNA-mediated gene knockdown

Human siRNAs were purchased from Dharmacon (Dharmacon, Lafayette, CO, USA). Transient siRNA knockdown was achieved by reverse transfection of 50 nM single or SMARTpool siRNA in 96-wells format with 6000 (MDA-MB-231), 5000 (Hs578T), 7000 (MDA-MB-468), 8000 (HCC1806) or 10,000 (T47D and MCF7) cells/well using the transfection reagent INTERFERin (Polyplus, Illkirch, France) according to the manufacturer’s guidelines. For other well formats, cell numbers were adjusted according to the well surface area. Medium was refreshed after 20 h and transfected cells were used for various assays till 7 days after transfection. siKinasePool, a mix of 720 siRNAs targeting human kinases was used as a control siRNA (final concentration of 50 nM).

### Cell death assays

Cells transfected with siRNAs in 96-wells format were stained with 100 ng/ml Hoechst 33342 (Thermo Scientific) after which cells were incubated with 100 nM propidium iodide (PI) and 0.05% Annexin-V-Alexa633 6 h before imaging. Imaging was performed with a Nikon Eclipse TiE 2000 microscope fitted with a 37 °C/5% CO2 incubation chamber, a × 20 objective (0.75 NA, 1.00 WD) and a perfect focus system (PFS). Images were taken at 4 positions/well and analyzed using CellProfiler and R studio software. After imaging, plates were fixed with trichloroacetic acid (TCA) and later used for cell proliferation assays.

Caspase activity was measured 4 days after transfection using the Caspase-Glo 3/7 assay (Promega) according to the manufacturer’s instructions.

### Cell proliferation assays

For nuclei counting, cells in 96-wells plates were fixed using trichloroacetic acid and stained with 100 ng/ml Hoechst 33258 (Thermo Scientific) in water. Imaging was performed on a Nikon Eclipse TiE 2000 microscope fitted with a 10x objective (0.30 NA, 16 WD) and PFS using 8 × 8 stitching to cover the whole well surface area. The number of nuclei was quantified using CellProfiler (REF). Next to nuclei counting, the sulforhodamine B (SRB) colorimetric assay (Vichai et al. 2006) was used to determine the effect of knockdown and treatment on cell proliferation.

### Immunofluorescence

For nuclei imaging, transfected cells were fixed with 70 μl 1% paraformaldehyde (PFA) / 0.1% Trition-X100 in PBS. Cells were washed 3 times with 0.5% bovine serum albumin (BSA) in PBS, stained with 100 ng/ml Hoechst (Thermo Scientific) in PBS, washed once with 0.5% BSA in PBS and washed twice with PBS. For SUN2 staining cells were fixed with 70 μl 1% paraformaldehyde (PFA) / 0.1% Trition-X100 in PBS. Cells were 3 times washed with 0.5% BSA in PBS and stained with primary antibody O/N at 4 °C. Next, cells were washed 3 times with 0.5% BSA in PBS and stained with Alexa-561 conjugated together with 100 ng/ml Hoechst 333258 for 1 h at room temperature. Stained cells were washed once with 0.5% BSA in PBS and washed twice with PBS. For phospho-Histone H3 staining, cells were fixed in with 4% formaldehyde for 10 min at 37 °C. Formaldehyde was removed and 90% methanol in PBS was slowly added and incubated on ice for 30 min. Fixed cells were washed twice with 0.5% BSA in PBS, incubated with primary antibody O/N at 4 °C, washed 3 times with 0.5% BSA in PBS and incubated with Alexa-488 conjugated secondary antibody together with 100 ng/ml Hoechst 33258 for 1 h at room temperature. Stained cells were washed once with 0.5% BSA in PBS and washed twice with PBS. For all stainings, cells were imaged with a Nikon Eclipse TiE 2000 microscope with a perfect focus system. For nuclear and phospho-histone H3 staining a 20x objective was used. For SUN2 staining a 60x oil objective was used.

### Fluorescence-activated cell sorting

For fluorescence-activated cell sorting (FACS) analysis of cell cycle progression, transfections were performed in 48-wells format. Seventy-two hours after transfection, samples were collected in 1 mM EDTA in PBS. The cell pellet was resuspended in 80% ice cold ethanol and stored at − 20 °C. Cells were centrifuged (5 min, 1000 rpm, 4 °C) and rehydrated in 1 ml PBS for 15 min at room temperature (RT). After centrifugation, cells were stained with 3 μM DAPI and 0.1% Nonidet P-40 freshly added to staining buffer (100 μM Tris, 150 mM NaCl, 1 mM CaCl2, 0.5 mM MgCl_2_ in milliQ) for 15 min at RT. 10,000 and 5000 events were recorded for control and knockdown conditions, respectively. For FACS analysis of phospho-Histone H3 positive cells, transfections were performed in 6-wells format. As a control, cells were treated with 100 ng/ml nocodazole 24 h before sample collection. Seventy-two hours after transfection, medium, washes and trypsinized cells were collected, centrifuged (5 min, 1000 rpm, 4 °C) and resuspended in PBS. Cells were fixed by adding 16% formaldehyde till a final concentration of 4% formaldehyde was reached and incubated at 37 °C for 10 min. Cells were pelleted and permeabilized in 90% methanol for 30 min on ice. The pellet was washed twice with 0.5% BSA in PBS, incubated with primary antibody for 1 h at RT, washed twice with 0.5% BSA in PBS, incubated with conjugated Alexa-488 secondary antibody for 30 min at RT, washed with 0.5% BSA in PBS and resuspended in PBS. All FACS samples were measured using BD FACSCanto II TM (BD Bioscience) and analysis was performed using FlowJo software.

### RNA isolation and cDNA synthesis

Twenty-four, forty-eight or seventy-two hours after transfection, total RNA was extracted using RNeasy plus mini kit (Qiagen) followed by cDNA synthesis using the RevertAid H minus first strand cDNA synthesis kit (Thermo Fisher Scientific) both according to the manufacturer’s protocol.

### RT-qPCR

RT-qPCR was performed with the SYBR Green PCR master mix (Thermo Fisher Scientific) on a 7500 Fast Real-Time PCR machine (Applied Biosystems/Thermo Fisher Scientific). Relative gene expression was calculated after correction for GAPDH and β-actin expression using the 2ΔΔCt method.

### PCR and gel electrophoresis

PCR was performed using the MyTaq Red Mix (Bioline) according to the manufacturer’s instructions. Products were loaded on a 0.8 or 2.0% agarose gel and visualized by using a transilluminator. Bands were quantified using ImageQuant software.

### Western blotting

Samples were lysed in RIPA lysis buffer (1% w/w deoxycholate, 50 mM Tris (pH 7.5), 0.15 M NaCl, 0.1% sodium dodecyl sulfate (SDS), 1% v/v NP-40, 2 mM EDTA, 1% v/v protease inhibitor cocktail (P8340, Sigma-Aldrich)) 72 h after transfection Proteins were separated by electrophoresis using SDS-PAGE gels, followed by transfer to PVDF membranes (Merck Millipore), blocked in 5% w/v BSA or milk and overnight incubated with the corresponding primary antibody at 4 °C. Membranes were incubated with secondary antibody for 1 h at room temperature, exposed to Pierce ECL western blotting substrate (Thermo Fisher Scientific) and visualized by using the Amersham Imager 600 (GE Healthcare). At least 2 biological replicates were performed per experiment. Tubulin was used as a loading control.

### mScarlet plasmid cloning and transfection

The pmScarlet_C1 plasmid (Plasmid #85042, Addgene) was used as a backbone. Total RNA was extracted from Hs578T cells using RNeasy plus mini kit (Qiagen) followed by cDNA synthesis using the RevertAid H minus first strand cDNA synthesis kit (Thermo Fisher Scientific) both according to the manufacturer’s protocol. To enrich for the CDCA5 intron retained product, the procedure was repeated 3 days after NHP2L1 knockdown. CDCA5 was amplified using the NEBQ5 hotstart master mix (New England Biolabs) using restriction site overhang primers (Forward: 5′-TAAGCAGAATTCATCTGGGAGGCGAACGC-3′, Reverse: 5′-TGCTTAGGATCCTCATTCAACCAGGAGATCAAACTGC-3′). PCR products were purified using the Wizard SV Gel and PCR Clean-Up System (Promega). CDCA5 inserts and pmScarlet_C1 backbone were enzymatically cleaved using EcoRI-HF and BamHI restriction enzymes (New England Biolabs), loaded on a agarose gel and purified with the Wizard SV Gel PCR Clean-Up System (Promega). Inserts and backbones were ligated using T7 DNA ligase (New England Biolabs) followed by transformation in C2987I electrocompetent bacteria (New England Biolabs). Colonies were screened for the desired product and validated by Sanger sequencing. Transfections were performed in MDA-MB-231 using Lipfectamine 3000 (Thermo Fisher) according to the manufacturer’s protocol.

### Immunoprecipitation

BAC reporter cell lines were plated in two T175s. Three days after cell plating, cells were trypsinized, resuspended in full RPMI washed with PBS twice and lysed in 0.3 ml EBC buffer (50 mM Tris pH 7.3, 150 mM NaCl, 0.5% NP-40, 1 mM MgCl2, 1% v/v protease inhibitor cocktail (P8340, Sigma-Aldrich)). Lysates were sonicated 6 times for 10 s, 500 U benzonase (E1014, Sigma) in 0.7 ml EBC was added and samples were incubated for 1 h at 4 °C under rotation. NaCl was added to a final concentration of 150 mM, lysates were spinned down for 10 min at full speed and lysates were added to 20 μl GFP-Trap A beads suspension (Chromotek) and uncubated for 1.5 h at 4 °C under rotation. For western blot, beads were washed 6 times with 1 ml EBC buffer containing 150 mM NaCl. 20 μl 2x sample buffer was added to the beads and the sample was boiled for 10 min at 95 °C before loading. For mass spectrometry, beads were washed twice with 1 ml EBC buffer containing 150 mM NaCl and twice with 1 ml 50 mM ammonium bicarbonate. Next, beads were O/N incubated with 250 μl 50 mM ammonium bicarbonate containing 2.5 μg trypsin (V5111, Promega) at 37 °C. Overnight digestion was stopped by adding 25% TFA (40,967, Sigma) till a final concentration of 1%. Digests were centrifuged for 5 min and loaded on a prepared tC18 cartridge (twice washing with acetonitrile, twice washing with 0.1% acetic acid). Peptides were desalted by washing twice with 0.1% acetic acid, after which the peptides were eluted with 0.1% acetic acid/60% acetonitrile.

### TCGA correlations

Normalized RNA sequencing data from The Cancer Genome Atlas (TCGA) was obtained by using the TCGA Assembler R package [16] after the new release in January 2017. Normalized reads were log2 transformed for both the correlation calculations as differences in expression levels between ER positive and TNBC tumors.

### Statistical analysis

Sample sizes were based on previously published similar experiments. When not indicated, all experiments were performed in biological triplicates. Significance was determined using a Student’s t-test (two-tailed, equal variances) or one-way ANOVA (for comparison of more than 2 groups) using GraphPad Prism 6.0. Results were considered to be significant if *p*-value < 0.05.

## Results

### Spliceosome proliferation screen in TNBC identifies splicing factors affecting cell cycle progression

**Fig. 1:**
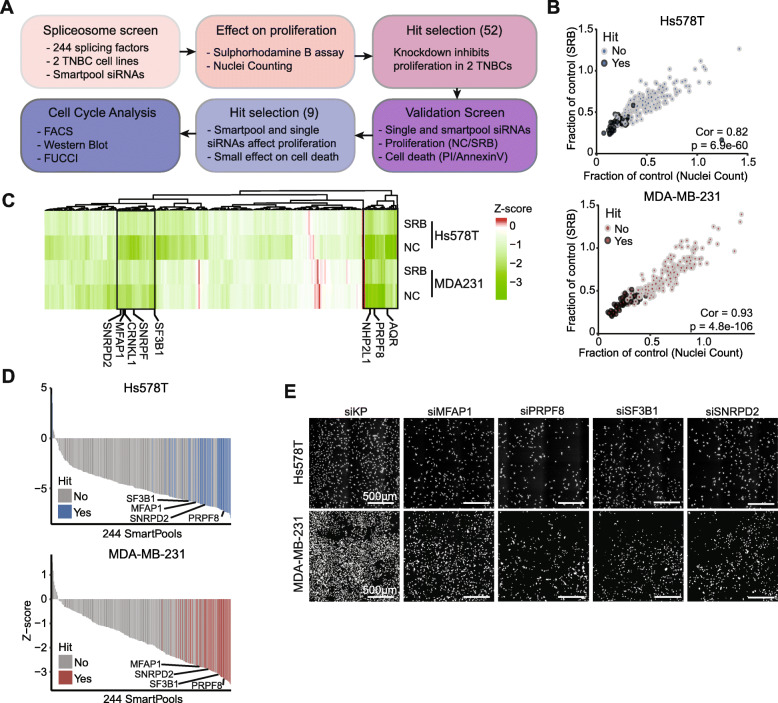
Spliceosome RNAi screen identifies novel splicing regulators of tumor cell proliferation*.* **a** Overview of steps used to select splicing factors involved in breast cancer cell proliferation. All assays were performed 72 h after transfection. **b** Correlation between SRB and nuclei count values (both proliferation measurements) in the primary screen for Hs578T (top) and MDA-MB-231 (bottom) cell lines. **c** Heatmap showing the Z-scores for splicing factor knockdown effects on proliferation in Hs578T and MDA-MB-231 72 h after knockdown. Splicing factors that upon knockdown inhibited proliferation in both assays and cell lines (highlighted clusters) were selected for further validation. **d** Nuclei counting Z-scores for all splicing factors in the primary screen. Factors selected for validation are highlighted in blue and red, respectively. **e** Examples of splicing factor knockdowns and nuclei counting. siKinasePool (siKP) was used as a negative control. Scale bar = 500 μm

**Fig. 2:**
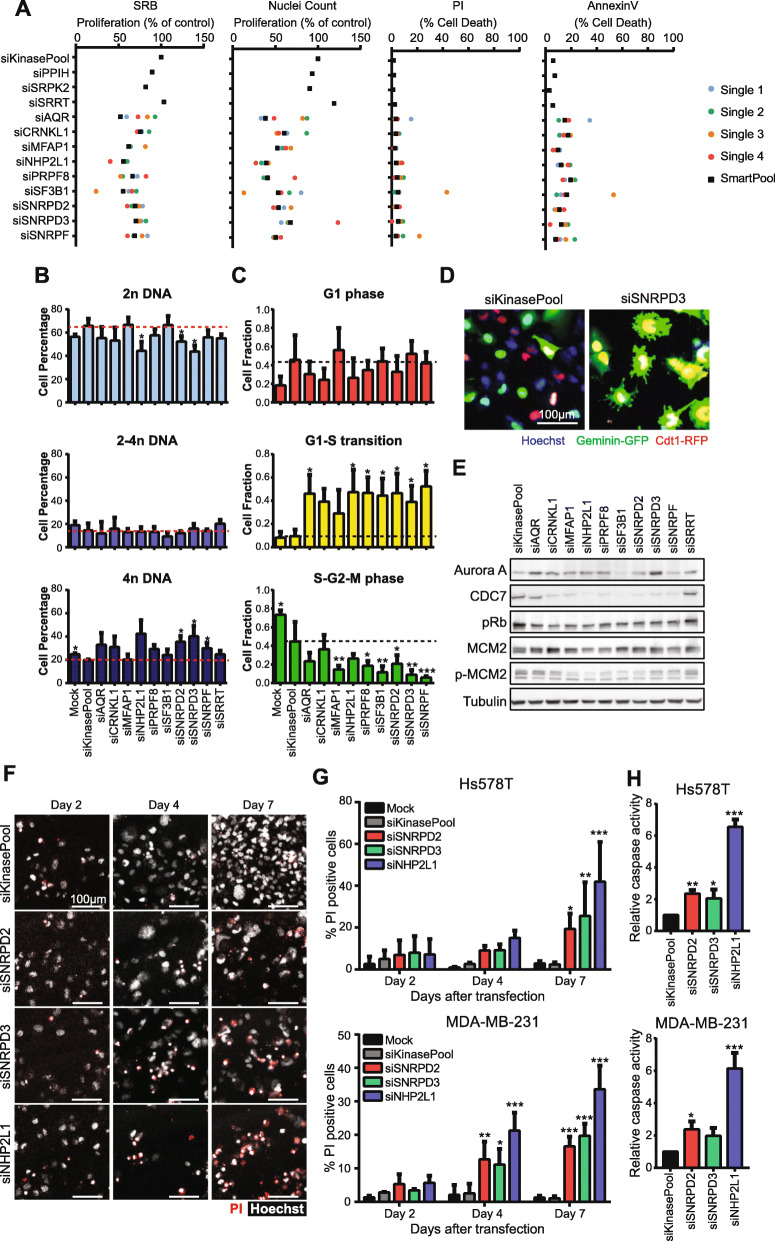
Effect of candidate splicing factor knockdown on cell death and cell cycle progression*.* **a** Results of validation screen; effect of SMARTpool and 4 single siRNAs of selected candidates on proliferation (SRB and nuclei count) and cell death (Annexin V and PI staining) in Hs578T cells 72 h after knockdown. PPIH, SRPK2 and SRRT were splicing factors not affecting proliferation in the primary screen and used as negative control. **b** Effect of splicing factor knockdown on cell DNA content in Hs578T cells measured by FACS analysis 72 h after knockdown. Mean + stdev of three biological replicates. siSRRT was used as a splicing factor negative control. **c** Effect of selected splicing factor knockdown on cell cycle arrest measured using the FUCCI cell cycle system in Hs578T cells 72 h after knockdown. Mean + stdev of three biological replicates. siSRRT was used as a splicing factor negative control. **d** Example of the effect of splicing factor SNRPD3 knockdown on FUCCI cell cycle markers in Hs578T cells. **e** Effect of splicing factor knockdown on expression levels of cell cycle regulators in Hs578T cells. Representative blots of two biological replicates. **f** Representative images of propidium iodide (PI) and Hoechst staining 2, 4 and 7 days after knockdown in Hs578T cells. Scale bar = 100 μm. **g** Percentage of cell death 2, 4 and 7 days after splicing factor knockdown in Hs578T (top) and MDA-MB-231 (bottom) cell lines. Mean + stdev of three biological replicates. **h** Effect of splicing factor knockdown on caspase activity in Hs578T (top) and MDA-MB-231 (bottom) cell lines 4 days after transfection. Mean + stdev of three biological replicates. Statistical significance was determined using ANOVA correcting for multiple testing. * *p* < 0.05, ** *p* < 0.01, *** *p* < 0.001

To systematically unravel which spliceosomal components may promote TNBC cell proliferation, we performed a RNAi screen for 244 splicing factors in two highly proliferative TNBC basal B cell lines: Hs578T and MDA-MB-231. Cells were transfected with SMARTpool siRNAs (pool of 4 single siRNAs per target) and proliferation was assessed using both sulphorhodamine B (SRB) assays [17] and nuclei counting (Fig. 1a); these two read-outs were highly correlated in both cell lines (correlation > 0.8, Fig. 1b). Clustering the spliceosomal components based on their modulation of proliferation using Z-scores revealed two clusters of in total 52 splicing factors that upon knockdown strongly reduced cell growth in both cell lines and assays (Fig. 1c-e, Suppl. Figure 1). Next, to eliminate components of which reduced levels induces cytotoxicity, we performed a validation RNAi screen in which we measured cell death in parallel with cell count. Ten splicing factors were selected of which depletion strongly inhibited cell growth with only limited induction of cell death 72 h after knockdown (Suppl. Figure 2A). These effects were validated for nine of these factors in a second biological replicate (Suppl. Figure 2B) and for all of these components, at least 2 of the single siRNAs demonstrated similar effects as the SMARTpool in both Hs578T and MDA-MB-231 cell lines (Fig. 2a, Suppl. Figure 2C). Remarkably, these nine splicing factors were all part of the core spliceosome (Suppl. Figure 3A), but distributed over different sub-complexes (Suppl. Figure 3B) [9]. Among our candidates was SF3B1, a splicing factor known to be a driver gene in breast cancer [18] and often mutated in uveal melanoma and chronic lymphocytic leukemia (CLL) [19, 20]. Knockdown of many of these nine candidates resulted in a higher percentage of cells containing 4n DNA in both TNBC cell lines (Fig. 2b, Suppl. Figure 4A). FUCCI imaging-based cell cycle analysis [21] demonstrated that splicing factor knockdown resulted in an increased cell fraction in G1-S transition accompanied by loss of cells in S-G2-M phase in Hs578T cells (Fig. 2c-d). In conjunction, splicing factor knockdown also led to decreased levels of CDC7, a regulator of G1/S transition, and the phosphorylation of its downstream target p-MCM2 (Fig. 2e, Suppl. Figure 4B). Altogether, we selected nine splicing factors which depletion resulted in a strong decrease in cell proliferation and cell cycle arrest in G1-S phase with nuclei containing 4n DNA content. Three of these factors, SNRPD2, SNRPD3 and NHP2L1, demonstrated strong effects on all of these aspects and were selected for further mechanistic studies.

**Fig. 3:**
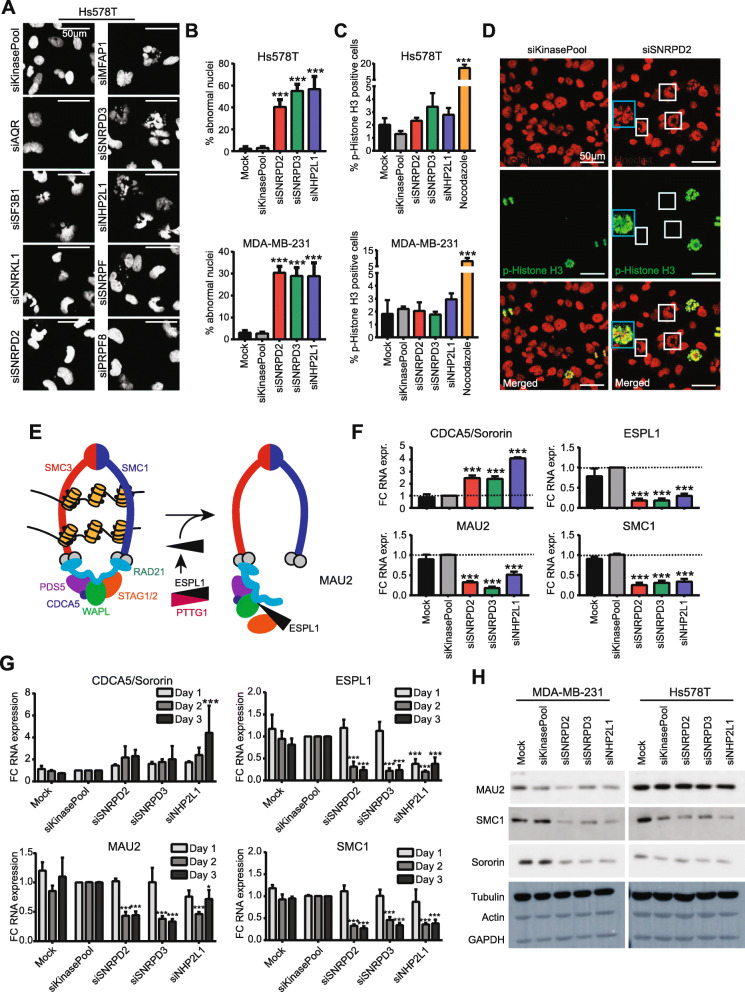
Effect of candidate splicing factor knockdown on sister chromatid cohesion*.* **a** Effect of splicing factor knockdown on nuclear phenotype in Hs578T cells 72 h after knockdown. Scale bar = 50 μm. **b** Percentage of cells displaying an abnormal nuclear phenotype 72 h after knockdown. Mean + stdev of at least 3 positions. **c** Percentage of p-Histone H3 positive cells upon splicing factor knockdown 72 h after knockdown. Nocodazole treatment was used as a positive control. Mean + stdev of 3 biological replicates. **d** Nuclear phenotype of p-histone H3 positive cells upon SNRPD2 knockdown in MDA-MB-231. Some cells with irregular nuclear phenotype are p-histone H3 positive (blue squares), while some are negative (white squares). Scale bar = 50 μm. **e** Overview of the factors involved in sister chromatid cohesion. Adapted from Peters et al, 2012. **f** Effect of SNRPD2, SNRPD3 and NHP2L1 knockdown on RNA expression levels of sororin, ESPL1, MAU2 and SMC1 in Hs578T 72 h after knockdown. Mean + stdev of 3 biological replicates. **g** Effect of SNRPD2, SNRPD3 and NHP2L1 knockdown on sororin, ESPL1, MAU2 and SMC1 expression levels 1, 2 and 3 days after knockdown in Hs578T. Mean + stdev of three biological replicates. (H) Protein MAU2, SMC1 and sororin levels 3 days after knockdown of SNRPD2, SNRPD3 and NHP2L1. Statistical significance was determined using ANOVA correcting for multiple testing. * *p* < 0.05, ** *p* < 0.01, *** *p* < 0.001

To investigate whether proliferative defects induced by loss of SNRPD2, SNRPD3 and NHP2L1 were TNBC and/or cell line specific, we performed knockdowns of these factors in four additional highly proliferative breast cancer cell lines covering different breast cancer subtypes (MDA-MB-468 and HCC1806 are from the TNBC basal A subtype, while T47D and MCF7 are from the luminal subtype). All cell lines except T47D demonstrated decreased cell growth and a slight increase in cell death upon splicing factor knockdown (Suppl. Figure 5A-D). This lower sensitivity of T47D cells could probably be explained by the lower proliferation rate of this cell line. In accordance with the TNBC basal B cell lines (Fig. 3), the strongest effects were observed for NHP2L1 knockdown.

Since SNRPD2, SNRPD3 and NHP2L1 knockdown halted proliferation due to G1-S arrest and showed the irregular nuclear phenotype, we hypothesized that knockdown of these splicing factors would lead to cell death on the long term. Accordingly, depletion of SNRPD2, SNRPD3 and NHP2L1 significantly induced cell death within 7 days after knockdown in Hs578T and within 4 days after knockdown in MDA-MB-231 cells (Fig. 2f-g, Suppl. Figure 6A-B). As expected, this was accompanied by an even stronger reduction of cell number (Suppl. Figure 6B). The increased cell death was mediated by apoptosis, demonstrated by the significant increase in caspase activity upon splicing factor knockdown (Fig. 2h).

### Depletion of SNRPD2, SNRPD3 and NHP2L1 affect sister chromatid cohesion

The increased number of cells bearing 4n DNA content (Fig. 2b, Suppl. Figure 4A) suggested a role for our splicing factors in mitosis related to breast cancer cell proliferation. A recent functional genomics screen identified some splicing factors as regulators of mitosis in HeLa cells [10]. In our screen, we systematically analyzed every single component of the spliceosome in TNBC cells: only 19 out of the 244 splicing factors were examined in HeLa cells. Yet for these 19 factors, a significant correlation between the percentage of abnormal nuclei in HeLa and proliferative defects was observed in MDA-MB-231, but not in Hs578T (Suppl. Figure 7). This suggests that at least in MDA-MB-231 cells, the observed proliferative defect upon splicing factor knockdown could be mediated by mitotic defects. Interestingly, knockdown of all of nine selected splicing factors demonstrated an increase in poly-lobed irregular shaped nuclei, a marker for abnormal mitosis (Fig. 3a-b, Suppl. Figure 8A). Moreover, knockdown of SNRPD2, SNRPD3 and NHP2L1 in other breast cancer cell lines also resulted in a similar irregular shaped nuclear phenotype (Suppl. Figure 8B), suggesting that cell growth inhibition through splicing factor knockdown is mediated by a common mechanism irrespective of the BC subtype. Knockdown of SNRPD2, SNRPD3 and NHP2L1 did not affect the percentage of cells positive for the mitosis marker p-Histone H3 (Fig. 3c). However, we observed a clear correlation between the irregular nuclear phenotype and expression of this marker (Fig. 3d, Suppl. Figure 9, blue squares). Since not all irregular shaped nuclei were positive for this mitotic marker (Fig. 3d, Suppl. Figure 9, white squares), we suggest the phenotype to arise during mitosis by incorrect cell division. Cells then proceed to G1 with 4n DNA and are halted during G1-S transition. Indeed, we observed that SNRPD2, SNRPD3 and NHP2L1 knockdown cells did spend longer in mitosis and did not distribute the DNA over the two daughter cells. Intriguingly, our observed nuclear phenotype is highly similar to nuclear phenotypes observed as a consequence of defects in sister chromatid cohesion (SCC) [10, 22]. The SCC complex forms a ring structure that holds the newly synthesized chromatid pairs together from replication in S-phase until mitosis and is thereby essential for chromosome orientation and segregation [23]. This ring structure consists of seven major components (SMC1, SMC3, RAD21, STAG1, STAG2, WAPL and PDS5) that can open and close before DNA replication during G1 phase. During DNA replication the two sister chromatids are captured within the ring structure, that is now stabilized by sororin (gene name CDCA5) that prevents it from opening [23]. During mitosis, ESPL1 cuts the ring structure open followed by distribution of the sister chromatids over the daughter cells (Fig. 3e, Suppl. Figure 10A). Systematic evaluation of the changes in RNA expression levels of these sister chromatid cohesion factors demonstrated a consistent downregulation of ESPL1, SMC1 and MAU2 and upregulation of sororin levels upon SNRPD2, SNRPD3 and NHP2L1 knockdown in both MDA-MB-231 and Hs578T cell lines (Fig. 3f, Suppl. Figure 10B-C). These changes in RNA expression levels already appeared within 2 days upon siRNA transfection (Fig. 3g, Suppl. Figure 10D). Remarkably, whereas sororin RNA levels were upregulated upon splicing factor knockdown, we observed decreased sororin protein levels (Fig. 3h). Altogether, we demonstrated that depletion of splicing factors SNRPD2, SNRPD3 and NHP2L1 affects sister chromatid cohesion factors sororin, ESPL1, SMC1 and MAU2 both in Hs578T and MDA-MB-231 cell lines in close association with impaired mitosis.

### Depletion of splicing components favors sororin intron retention and reduced sororin protein levels

**Fig. 4:**
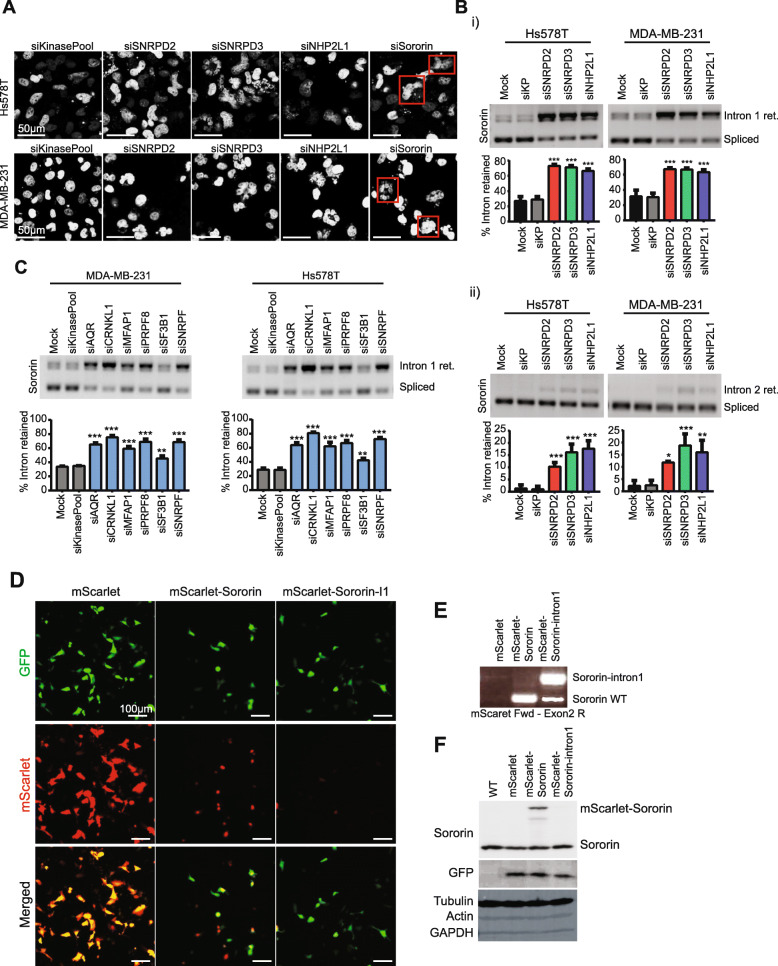
Candidate splicing factor knockdown results in sororin intron 1 retention leading to reduction of sororin protein levels*.* a Nuclear phenotype 72 h after candidate splicing factor or sororin knockdown in MDA-MB-231 and Hs578T cells. Scale bar = 50 μm. **b** Sororin intron 1 (i) and intron 2 (ii) retention 72 h after SNRPD2, SNRPD3 and NHP2L1 knockdown in Hs578T and MDA-MB-231 cells. Mean + stdev of 3 biological replicates. **c** Sororin intron 1 retention 72 h after knockdown of other splicing factor candidates in Hs578T and MDA-MB-231 cells. Mean + stdev of 3 biological replicates. **d** Confocal images of MDA-MB-231 cells 24 h after transfection with mScarlet, mScarlet tagged sororin (without introns) or mScarlet tagged sororin with intron 1 retained. mScarlet transfections were combined with GFP vectors to control for transfection efficiency. Scale bar = 100 μm. **e** Alternative splicing of sororin 24 h after transfection with different mScarlet-Sororin plasmids. Forward primer bound to mScarlet, reversed primer bound to sororin exon 2. **f** Sororin protein expression 24 h after co-transfection with mScarlet and GFP plasmids. Statistical significance was determined using ANOVA correcting for multiple testing. * *p* < 0.05, ** *p* < 0.01, *** *p* < 0.001

We previously showed that knockdown of SNRPD2, SNRPD3 and NHP2L1 induced upregulation of sororin RNA expression (Fig. 3f, Suppl. Figure 10C), while sororin protein level was downregulated (Fig. 3h). Moreover, knockdown of sororin could recapitulate the nuclear phenotype observed upon splicing factor knockdown (Fig. 4a), suggesting that sororin could be the cause of these observed defects in TNBC cell proliferation. Previous studies demonstrated that sororin can be alternatively spliced upon splicing factor depletion; mostly by changes in intron 1, 2 and 5 retention [10, 11]. Interestingly, SNRPD2, SNPRD3 and NHP2L1 knockdown consistently enhanced intron 1 and/or intron 2 retention in both TNBC cell lines (Suppl. Figure 11A), while intron 5 retention was cell line and knockdown dependent (Suppl. Figure 11B). Increased sororin intron 1 and/or 2 retention already appeared 24 h after transfection (Suppl. Figure 11C), confirming that sororin could be a direct mRNA target of SNRPD2, SNRPD3 and NHP2L1. Analysis of the separate introns revealed that both intron 1 and 2 were retained upon splicing factor knockdown in both TNBC cell lines, while the other introns were not affected (Fig. 4b, Suppl. Figure 12A-C). Next, we investigated whether this effect was also observed for the other splicing candidates discovered in our proliferation screen. Indeed, depletion of all of these factors significantly increased intron 1 retention in both MDA-MB-231 and Hs578T cells (Fig. 4c). Enhanced intron 2 retention was only observed after CRNKL1, PRPF8, SF3B1 and SNRPF depletion (Suppl. Figure 12D), suggesting that the first intron is commonly spliced by this set of splicing factors. Altogether, we can conclude that we identified a group of splicing factors which depletion arrests the cell cycle through a common transcriptional mechanism that results in increased sororin intron 1 retention. To corroborate that the increased intron 1 retention results in reduced protein levels, we generated a sororin-mScarlet fusion plasmid for both wild type and intron 1 retained sororin (sororin-Intron1). Although the intron could be spliced out, transfection with sororin-intron1 plasmid mainly resulted in intron retained RNA transcripts (Fig. 4e). While transfection with the sororin wild type plasmid resulted in sororin protein expression, translation was less efficient for the sororin-intron1 plasmid (Fig. 4d and f) confirming that intron 1 retention contributes to reduced protein levels.

### SUN2 interacts with SNRPD2, SNPRD3 and NHP2L1 splicing factors and modulates sororin splicing and SCC

**Fig. 5:**
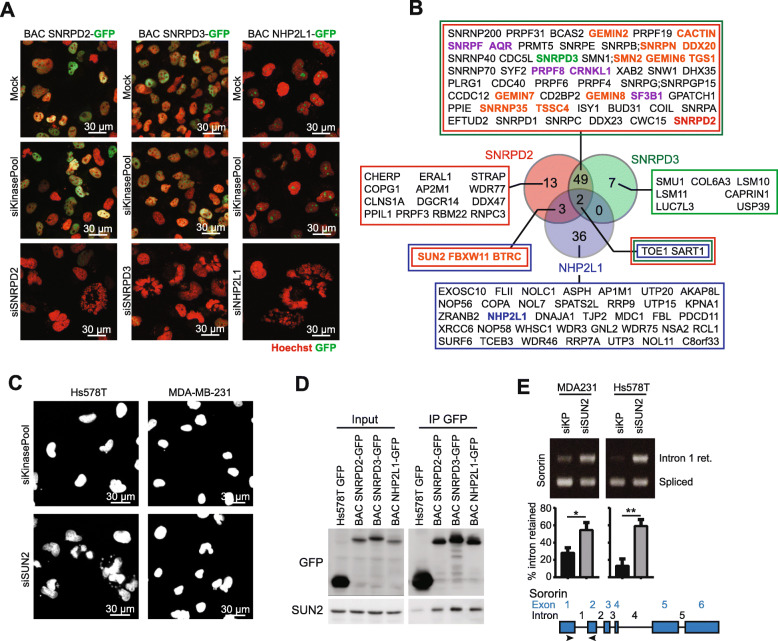
SNRPD2, SNRPD3 and NHP2L1 protein interactions. **a** Characterization of Hs578T GFP BAC-reporter cell lines for splicing factors SNRPD2, SNRPD3 and NHP2L1. Images were captured 72 h after transfection. Scale bar = 30 μm. **b** Overlap of proteins residing in SNRPD2, SNRPD3 and NHP2L1 complexes, respectively. Splicing factors belonging to the nine candidates selected from the primary screen are labeled in purple. Orange: proteins that were selected for further validation. **c** Nuclear phenotype 72 h after SUN2 knockdown in Hs578T and MDA-MB-231 cells. Scale bar = 30 μm. **d** Co-immunoprecipitation of SNRPD2, SNRPD3 and NHP2L1 Hs578T BAC GFP-reporters with SUN2. **e** Alternative splicing of sororin intron 1 upon SUN2 knockdown. Experiments were performed in biological triplicates. Significance was determined using a Student’s T-test. * *p* < 0.05, ** *p* < 0.01

We further focused on SNRPD2, SNRPD3 and NHP2L1, and evaluated their protein complex. For this we established bacterial artificial chromosome (BAC) green fluorescent protein (GFP) reporter cell lines for these three splicing factors, allowing endogenous regulation of expression of GFP-fusion products. Although the effects of the GFP tag on the spatial confirmation cannot be excluded, expected localization of all splicing factors in the nuclei was observed. Interestingly, SNRPD2 and SNRPD3 showed similar speckled pattern in the nucleus, while NHP2L1 showed a specific localization resembling nucleoli. Knockdown of SNRPD2, SNRPD3 or NHP2L1 reduced the GFP intensity (Fig. 5a, Suppl. Figure 13A-B), supporting expression of correct GFP-fusions proteins. To identify the functional partners of SNRPD2, SNPRD3 and NHP2L1, we performed GFP pulldown followed by mass spectrometry proteomics on the three different GFP-fusion cell lines. SRNDP2 and SNRPD3 were part of the same complex and showed 51 commonly co-immunoprecipitated proteins (Fig. 5b, Supplemental Table 1). Interestingly, seven out of the nine splicing factors identified in our initial screen resided in this complex, including AQR, SNRPF, PRPF8, CRNKL1 and SF3B1 (purple labeled in Fig. 5b), further supporting the probably limited effects of the GFP tag on the splicing factor interactomes. Pathway overrepresentation analysis of proteins in the different complexes demonstrated that SNRPD2 and SNRPD3 were mainly in a complex with other factors involved in splicing and RNA processing, while the NHP2L1 complex was enriched for factors involved in ribosomal RNA biogenesis and processing that are known to occur in the nucleoli (Suppl. Figures 14 and 15). To uncover splicing factor-interacting proteins that play a role in the previously observed reduced proliferation, we examined the nuclear phenotype of proteins interacting with at least two splicing factors and that were not part of our primary screen (Fig. 5b, orange names, Suppl. Figure 16). Intriguingly, depletion of nine of the fourteen tested genes including GEMIN6 and SNRNP35, showed a similar irregular nuclear phenotype as observed upon SNRPD2, SNRPD3 or NHP2L1 knockdown. Many of these nine genes have been related to splicing; CACTIN [24] and SMN2 [25] demonstrated a direct role in RNA splicing and gemins are known to be involved in spliceomome assembly [26]. Of specific interest was SUN2 (Fig. 5c), a protein that is part of the Linkers of Nucleoskeleton and Cytoskeleton (LINC) complexes connecting the nuclear to the cytoplasmic cytoskeleton and mainly resides in the nuclear lamina [27, 28]. Interestingly, SUN2 has also been related to mitosis in various ways: 1) SUN2 forms a physical interaction between the nuclear envelope and the centrosome [29], 2) SUN1 and SUN2 contribute to normal meiosis in *Arabidopsis thaliana* [30] and 3) SUN2 proteins regulate mitotic spindle orientation and mitotic progression [31]. SUN2 was significantly enriched in the SNRPD2 and NHP2L1 complexes, while almost significant enrichment was observed in the SNRPD3 complex (Supplemental Table 1) suggesting that SUN2 could interact with all of these splicing factors. Indeed, we validated the SUN2 interaction with SNRPD2, SNRPD3 and NHP2L1 (Fig. 5d). Importantly, SUN2 knockdown also increased sororin intron 1 retention similarly as observed after depletion of SNRPD2, SNRPD3 and NHP2L1 (Fig. 5e), without affecting intron 2 retention (Suppl. Figure 17). Altogether, we have evidence that our identified splicing factors affecting TNBC proliferation mainly function in the same complex containing many other splicing factors, but also interact with proteins known to play a critical role in mitosis such as SUN2 confirming an important interplay between splicing and mitosis.

### Pharmacological modulation of splicing factors affects sororin retention, SCC and TNBC survival

**Fig. 6:**
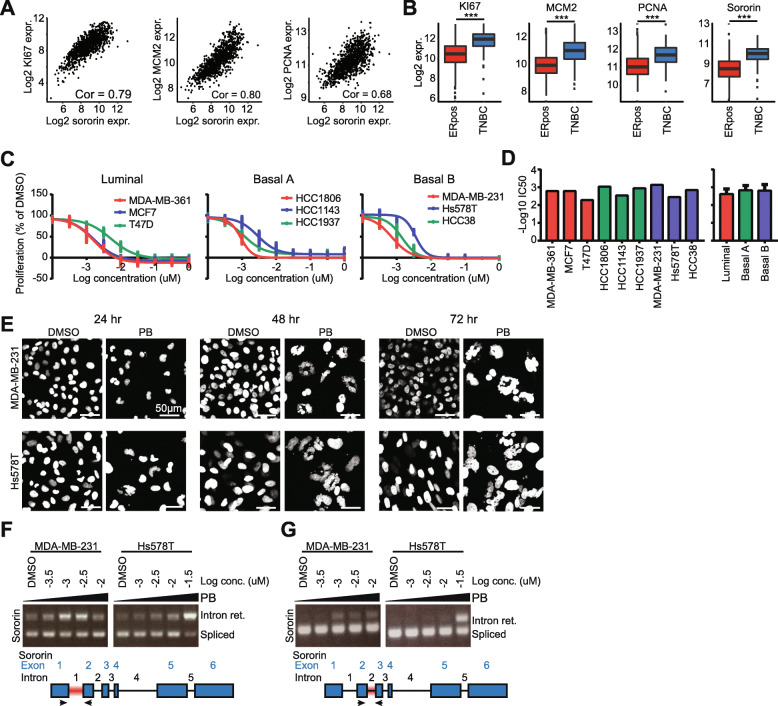
Effect of Pladienolide B treatment on breast cancer cell proliferation*.* **a** Correlation between CDCA5 and KI67, MCM2 and PCNA expression in 1097 primary breast tumors. **b** Log2 RNA expression levels of KI67, MCM2, PCNA and CDCA5 in ER positive (*n* = 807) and TNBC (*n* = 116) primary breast tumors. Statistical significance was determined using a Students’s t-test. *** *p* < 0.001. **c** Effect of 4 days PB treatment on proliferation of luminal, basal A or basal B cell lines. Mean + sd of 3 biological replicates. **d** PB treatment IC50 in luminal, basal A and basal B cell lines. **e** Effect of PB treatment (0.003uM) on MDA-MB-231 and Hs578T nuclear phenotype 24, 48 and 72 h after treatment. Scale bar = 50 um. **f** CDCA5 intron 1 retention after 48 h PB treatment. **g** CDCA5 intron 2 retention after 48 h PB treatment

Finally, we determined a strategy to pharmacologically modulate the TNBC splicing program and derive a similar mitotic phenotype as with depleting our key splicing factors. Given the overall critical connection between BC cell proliferation and correct splicing of sororin, we anticipated that overall sororin expression could be an indicative biomarker for highly proliferative human breast cancers. Indeed, sororin RNA expression levels were highly related to the expression levels of genuinely used proliferation markers such as Ki67, PCNA and MCM2 in human patient tumors (Fig. 6a), with all of these markers being higher expressed in TNBC compared to ER positive tumors (Fig. 6b). Various splicing factor inhibitors have been developed and the vast majority of these inhibitors, including plandienolide B (PB), targets SF3B1. Since SF3B1 was one of the nine splicing candidates for which depletion strongly inhibited cell proliferation in TNBC we evaluated whether PB could effectively inhibit proliferation of breast cancer cells. PB showed potent anti-proliferative effects in various breast cancer cell lines with an overall IC50 of almost 1 nM (Fig. 6c-d). PB treatment resulted in a similar nuclear phenotype as observed for the various splicing factors that did affect SCC, including SF3B1 (Fig. 6e and see Fig. 3a and 4a); this phenotype already appeared 24 h after treatment, but became more evident after 48 and 72 h of PB treatment (Fig. 6e). As anticiptated, in conjunction with this phenotype PB treatment enhanced sororin intron 1 and 2 retention in MDA-MB-231 and Hs578T cell lines (Fig. 6f-g). Altogether, this indicates that decreasing sororin levels through pharmacologically targeting the splicing machinery could be an interesting strategy to combat TNBC progression in breast cancer patients.

## Discussion

**Fig. 7:**
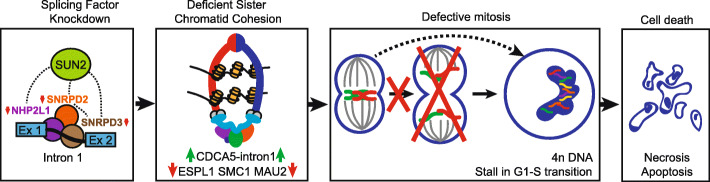
Role of candidate splicing factors in TNBC proliferation*.* Splicing factors knockdown leads to complex dysfunction, resulting in CDCA5 intron retention and downregulation of ESPL1, SMC1 and MAU2. Deficient sister chromatid cohesion results in misalignt chromosomes leading to incomplete mitosis, cells bearing double DNA content, stalling in G1-S transition and ultimately cell death

Accumulating evidence suggests that RNA splicing is critical in cancer cell proliferation in general and in breast cancer in particular [5–8, 10, 32–34]. Especially the role of many splicing factors in hormone receptor induced proliferation has been elucidated in the last decades [12, 13, 35–38]. However, the role of splicing factors in TNBC progression has not yet been evaluated. Here, through RNAi screening, we systematically evaluated the role of 244 splicing factors in TNBC proliferation and identified nine splicing factors that upon knockdown were consistently inhibiting proliferation in the Hs578T and MDA-MB-231 cell lines. Knockdown of these splicing factors resulted in defective sister chromatid cohesion due to increased sororin intron 1 retention and downregulation of ESPL1, MAU2 and SMC1. Consequently, cells bearing double DNA content, did stall in G1-S phase and ultimately underwent cell death (Fig. 7).

We have mapped the splicing factors that are critical in TNBC proliferation. Some of these splicing factors have been linked to progression of cancer. For example, SF3B1 was identified as a breast cancer driver gene [18], mutated in 20% of uveal melanoma tumors [39] and SF3B1 mutations have been related to adverse clinical outcome in CLL [19, 20]. Moreover, SNRPD2 and AQR knockdown inhibited proliferation in breast, pancreatic and ovarian cancer cell lines [40]. Interestingly, many of these splicing factors (AQR, MFAP1, NHP2L1, PRPF8, SF3B1, SNRPD3 and SNRPF) were identified to affect SCC in the cervical HeLa cancer cell line of which a limited number (NHP2L1, MFAP1 and PRPF8) was linked to sororin alternative splicing [10, 33], suggesting that the regulation of SCC by splicing factors is a common mechanism independent of the cancer type. Next to NHP2L1, MFAP1 and PRPF8, these studies also identified additional factors affecting sororin splicing (SART1, SKIIP, SF3A1, UBL5, CDC5L, U2AF2, BUD31) of which the majority (SART1, SKIIP, SF3A1 and UBL5) were also identified as TNBC proliferation regulators in our primary screen [10, 32–34]. However, U2AF2 depletion only demonstrated mild proliferative defects in our primary screen. We observed consistent downregulation of other SCC factors ESPL1, MAU2 and SMC1 upon splicing factor depletion, which was not observed by others [10], suggesting that there are cancer type-specific effects for some splicing factors. Taking into consideration the information of these previous studies and the very stringent criteria we used to select these nine splicing factors, we suggest that there are more splicing factors eliciting similar effects that might be potential targets to combat TNBC progression.

Using proteomics approaches to uncover the protein network associated with SNRPD2, SRNPD3 and NHP2L1, we discovered that seven out of our nine selected TNBC proliferation modulating splicing factors are present in the same protein-protein complex. Next to our candidates, this complex also contains additional splicing factors of which some (SNW1/SKIIP, CACTIN and BUD31) have already been shown to induce sororin intron 1 retention in different models [10, 11, 33], thus providing additional splicing factors that are of interest in relation to tumor cell proliferation. Interestingly, recently a direct interaction of splicing factors with SCC factors has been reported [41], suggesting that next to the indirect effects on SCC via sororin alternative splicing, splicing factors might also directly regulate SCC.

Our studies identified a novel common splicing factor interacting protein, SUN2, which is also essential for SCC and critical for proper sororin splicing. Such a role of SUN2 in splicing has not been described yet. SUN2 is part of the complex that links the nucleoskeleton to the cytoskeleton and locates in the nuclear envelope [42]. Additionally, the family of SUN proteins have also been linked to nucleolar morphology [43] and mitotic spindle orientation [31]. In our TNBC cell lines, SUN2 localized intranuclearly as well as in the nuclear envelope and was associated with different splicing factor containing complexes that modulate the SCC: SNRPD2 and NHP2L1 complexes. Further studies have to unravel how SUN2 interacts with these complexes and whether it is required for either the stability of the splicing complexes and/or their activity, and as such modulate the splicing of sororin and consequently ensure effective SCC.

In line with the increasing evidence for the role of splicing in cancer progression, the potential for inhibiting splicing factors as cancer treatment is broadly investigated. As discussed above; one of our splicing factor candidates was SF3B1, a commonly mutated gene in uveal melanoma and CLL and a potential target for cancer treatment. Pladienolide B has been discovered as a potent and specific SF3B1 inhibitor [44, 45] with anti-proliferative effects in CLL and gastric cancer [46, 47]. In the present study, we demonstrated that targeting SF3B1 using pladienolide B strongly affected breast cancer proliferation by increasing sororin alternative splicing and affecting the nuclear phenotype similar to our candidate splicing factor knockdown. Although the identified splicing factors were not differentially expressed between tumor and normal tissue or different breast cancer subtypes, we identified that RNA levels of downstream target sororin are highly correlated to proliferation markers in patient breast cancer tumors, suggesting that decreasing sororin levels after pladienolide B treatment or other pharmacological modulators of critical splicing factors could be a promising therapeutic avenue in the future treatment of breast cancer patients. This hypothesis is supported by the increased potency of Pladienolide B in cancer when compared to normal gastric cells [47]. Since sororin levels are significantly higher expressed in TNBC compared to hormone receptor positive tumors, we anticipate that pladienolide B treatment would in particularly be effective in the TNBC subtype. Further clinical studies should evaluate the value and adverse effects of pladienolide B and additional splicing inhibitors in TNBC treatment.

## Conclusion

In conclusion, we systematically determined splicing factors that control proliferation of breast cancer cells through a mechanism that involves effective sororin splicing and thereby appropriate SCC. SUN2 is an important new spliceosome complex interacting protein that is critically determining sororin splicing and SCC. Downregulation of the splicing factors, SUN2 or pharmacological inhibition of splicing leads to mitotic defects, G1-S arrest and ultimately cell death. We anticipate that targeting the various splicing targets in TNBC could be a highly effective strategy for anti-cancer drug development.

## Supplementary Information

**Additional file 1: Supplemental Figure 1.** Nuclei counting images of splicing factors inhibiting cell proliferation upon knockdown in both MDA-MB-231 and Hs578T 72 h after transfection*.* Scale bar = 500 μm. **Supplemental Figure 2.** Spliceosome RNAi validation screens*.* (A) Results of validation screen; effect of splicing factor knockdown on proliferation (SRB and nuclei count) and cell death (Annexin V and PI staining). Factors highlighted in green were selected for further validation. (B) Validation of candidate splicing factor SMARTpool knockdown on proliferation (SRB and Nuclei count) and cell death (PI and AnnexinV) in Hs578T (left) and MDA-MB-231 (right). Splicing factors selected for further validation are highlighted in green. (C) Validation of selected splicing factors using 4 single and SMARTpool siRNAs in MDA-MB-231. All measurements were performed 72 h after knockdown. **Supplemental Figure 3.** Spliceosome candidate distribution over different subcomplexes*.* (A) Candidate distribution over core and non-core complexes. (B) Candidate distributation of functional splicing subcomplexes. **Supplemental Figure 4.** Effect of candidate splicing factor knockdown on cell death and cell cycle progression in MDA-MB-231*.* (A) Effect of splicing factor knockdown on cell DNA content measured by FACS analysis in MDA-MB-231 cells 72 h after transfection. Mean + stdev of three biological replicates. SRRT is a splicing factor not affecting proliferation and was used as negative control. (B) Effect of splicing factor knockdown on expression levels of cell cycle regulators in MDA-MB-231 cells 72 h after transfection. Representative blots of two biological replicates. SRRT is a splicing factor not affecting proliferation and was used as negative control.Statistical significance was determined using ANOVA correcting for multiple testing. * *p* < 0.05, ** *p* < 0.01, *** *p* < 0.001. **Supplemenatal Figure 5.** Effect of SNRPD2, SNRPD3 and NHP2L1 knockdown in other cell lines*.* Effect of SNRPD2, SNRPD3 and NHP2L1 knockdown in proliferation and cell death three days after knockdown in HCC1806 (A), MDA-MB-468 (B), MCF7 (C) and T47D (D) cells. Mean + stdev of 3 biological replicates. Significance was determined using ANOVA correcting for multiple testing. **Supplemental Figure 6.** SNRPD2, SNRPD3 and NHP2L1 knockdown reduce proliferation and induce cell death*.* (A) Representative images of propidium iodide (PI) and Hoechst staining 2, 4 and 7 days after knockdown in MDA-MB-231 cells. Scale bar = 100 μm. (B) Cell number 2, 4 and 7 days after splicing factor knockdown in Hs578T (top) and MDA-MB-231 (bottom) cell lines. Cell growth was measured using the SRB absorbance. Mean + stdev of three biological replicates. Statistical significance was calculated using ANOVA correcting for multiple testing. * *p* < 0.05, ** *p* < 0.01, *** *p* < 0.001. **Supplemental Figure 7.** Correlation primary screen with mitotic screen Sundamoorthy et al, 2014*.* **Supplemenatal Figure 8.** Effect of SNRPD2, SNRPD3 and NHP2L1 on nuclear phenotype*.* (A) Nuclear phenotype 72 h after splicing factor knockdown in MDA-MB-231 cells. Scale bar = 50 μm. (B) Effect of SNRPD2, SNRPD3 and NHP2L1 knockdown on nuclear phenotype in HCC1806, MDA-MB-468, MCF7 and T47D cells 72 h after transfection. Scale bar = 20 μm. **Supplemental Figure 9.** Effect of SNRPD2, SNRPD3 and NHP2L1 knockdown on nuclear phenotype and mitosis*.* (A) Nuclear phenotype of p-histone H3 positive cells 72 h after SNRPD2, SNRPD3 or NHP2L1 knockdown in Hs578T. Some cells with irregular nuclear phenotype are p-histone H3 positive (white squares), while some are negative (blue squares). (B) Similar as A, but for MDA-MB-231. Scale bar = 100 μm. **Supplemenatal Figure 10.** Effect of candidate splicing factor knockdown on sister chromatid cohesion components*.* (A) Overview of the factors involved in sister chromatid cohesion. Adapted from Peters et al., 2012. (B) Effect of 72 h SNRPD2, SNRPD3 and NHP2L1 knockdown on RNA expression levels of genes involved in sister chromatid cohesion in Hs578T. Mean + sd of 3 biological replicates.(C) Effect of 72 h SNRPD2, SNRPD3 and NHP2L1 knockdown on RNA expression levels of genes involved in sister chromatid cohesion in MDA-MB-231. Mean + sd of 3 biological replicates. (D) Effect of SNRPD2, SNRPD3 and NHP2L1 knockdown on sororin, ESPL1, MAU2 and SMC1 expression levels 1, 2 and 3 days after knockdown in MDA-MB-231. Mean + sd of 3 biological replicates. Statistical significance was determined using ANOVA correcting for multiple testing. * *p* < 0.05, ** *p* < 0.01, *** *p* < 0.001. **Supplemental Figure 11.** Effect of SNRPD2, SNRPD3 and NHP2L1 knockdown on sororin intron retention*.* (A) Effect of 72 h SNRPD2, SNRPD3 and NHP2L1 knockdown on sororin intron 1 and 2 retention in Hs578T and MDA-MB-231 cells. Mean + sd of three biological replicates. (B) Effect of 72 h SNRPD2, SRNPD3 and NHP2L1 knockdown on sororin intron 5 retention in Hs578T and MDA-MB-231 cells. Mean + sd of three biological replicates. (C) Effect of 72 h SNRPD2, SNRPD3 and NHP2L1 knockdown on sororin intron 1 and 2 retention 1, 2 and 3 days after knockdown in Hs578T and MDA-MB-231. Mean + sd of three biological replicates. Statistical significance was determined using ANOVA correcting for multiple testing. * *p* < 0.05, ** *p* < 0.01, *** *p* < 0.001. **Supplemental Figure 12.** Effect of candidate splicing factor knockdown on sororin intron retention*.* Effect of SNRPD2, SNRPD3 and NHP2L1 knockdown on sororin intron 3 (A), intron 4 (B) or intron 5 (C) retention in Hs578T and MDA-MB-231 cell lines 72 h after transfection. (D) Effect of candidate splicing factor knockdown on sororin intron 2 retention in MDA-MB-231 and Hs578T cells 72 h after transfection. SRRT is a splicing factor not affecting TNBC proliferation and was used as a negative control. Mean + sd of three biological replicates. Mean + sd of three biological replicates. Statistical significance was determined using ANOVA correcting for multiple testing. * *p* < 0.05, ** *p* < 0.01, *** *p* < 0.001. **Supplemental Figure 13.** SNRPD2, SNRPD3 and NHP2L1 Hs578T BAC-GFP validation*.* (A) Endogenous and GFP fusion protein levels of SNRPD2, SNRPD3 and NHP2L1 in Hs578T BAC reporter cell lines 72 h after control and target knockdown. (B) Endogenous and GFP fusion protein levels in Hs578T wild type and BAC reporter cell lines. **Supplemental Figure 14.** Overrepresentation analysis of proteins in complex with SNRPD2, SNRPD3 or NHP2L1*.* Significantly enriched proteins were used for overrepresentation analysis using ConsensusPathDB (Kamburov) for Reactome and KEGG pathways. **Supplemental Figure 15**. Hierarchical clustering of proteins enriched in at least one of the splicing factor (SNRPD2, SNRPD3 or NHP2L1) complexes*.* From right to left; 1) involvement in commonly overrepresented pathways, 2) log2 fold change enrichment in splicing factor complex, 3) -log10 adjusted *P*-value for enrichment in splicing factor complex and 4) significance of the protein associated with the SNRPD2, SNRPD3 or NHP2L1 complex. **Supplemental Figure 16.** Nuclear phenotype upon knockdown of components in splicing factor complexes*.* Hoechst staining 72 h after transfection in Hs578T cells. Red labeled factors show abnormal nucleur phenotype upon knockdown. Scale bar = 100 μm. Right: zoom in of dashed square in left image. **Supplemental Figure 17.** Sororin intron 2 retention after SUN2 knockdown in MDA-MB-231 and Hs578T cell lines*.*

**Additional file 2.**

## Data Availability

The authors confirm that the data supporting the findings of this study are available within the article [and/or] its supplementary materials.

## Abbreviations

BAC: Bacterial artificial chromosome
CLL: Chronic lymphocytic leukemia
ER: Estrogen receptor
GFP: Green fluorescent protein
HER2: Human epidermal growth factor receptor 2
LINC: Linkers of nucleoskeleton and cytoskeleton
PB: Pladienolide B
PR: Progesteron receptor
SCC: Sister chromatid cohesion
snRNP: Small nuclear ribonucleoprotein
SRB: Sulphorhodamine B
TNBC: Triple negative breast cancer
