# Supplementary material for: Splicing factors control triple-negative breast cancer cell mitosis through SUN2 interaction and sororin intron retention
Source: J Exp Clin Cancer Res. 2021 Mar 1;40:82. doi: 10.1186/s13046-021-01863-4 (PMC7919097; doi:10.1186/s13046-021-01863-4)
Supplement: Supplementary file 1 — Additional file 1: Supplemental Figure 1. Nuclei counting images of splicing factors inhibiting cell proliferation upon knockdown in both MDA-MB-231 and Hs578T 72 h after transfection. Scale bar = 500 μm. Supplemental Figure 2. Spliceosome RNAi validation screens. (A) Results of validation screen; effect of splicing factor knockdown on proliferation (SRB and nuclei count) and cell death (Annexin V and PI staining). Factors highlighted in green were selected for further validation. (B) Validation of candidate splicing factor SMARTpool knockdown on proliferation (SRB and Nuclei count) and cell death (PI and AnnexinV) in Hs578T (left) and MDA-MB-231 (right). Splicing factors selected for further validation are highlighted in green. (C) Validation of selected splicing factors using 4 single and SMARTpool siRNAs in MDA-MB-231. All measurements were performed 72 h after knockdown. Supplemental Figure 3. Spliceosome candidate distribution over different subcomplexes. (A) Candidate distribution over core and non-core complexes. (B) Candidate distributation of functional splicing subcomplexes. Supplemental Figure 4. Effect of candidate splicing factor knockdown on cell death and cell cycle progression in MDA-MB-231. (A) Effect of splicing factor knockdown on cell DNA content measured by FACS analysis in MDA-MB-231 cells 72 h after transfection. Mean + stdev of three biological replicates. SRRT is a splicing factor not affecting proliferation and was used as negative control. (B) Effect of splicing factor knockdown on expression levels of cell cycle regulators in MDA-MB-231 cells 72 h after transfection. Representative blots of two biological replicates. SRRT is a splicing factor not affecting proliferation and was used as negative control.Statistical significance was determined using ANOVA correcting for multiple testing. * p < 0.05, ** p < 0.01, *** p < 0.001. Supplemenatal Figure 5. Effect of SNRPD2, SNRPD3 and NHP2L1 knockdown in other cell lines. Effect of SNRPD2, SNRPD3 [file 13046_2021_1863_MOESM1_ESM.pdf]

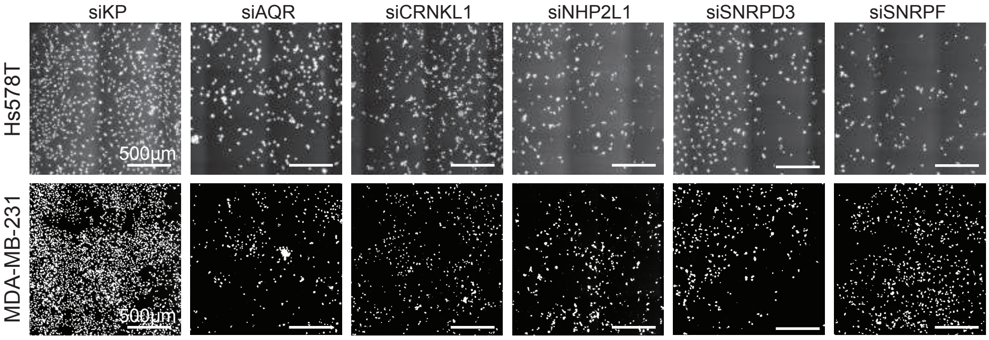

Supplemental Figure 1. ***Nuclei counting images of splicing factors inhibiting cell proliferation upon knockdown in both MDA-MB-231 and Hs578T 72 hours after transfection.*** Scale bar = 500µm.

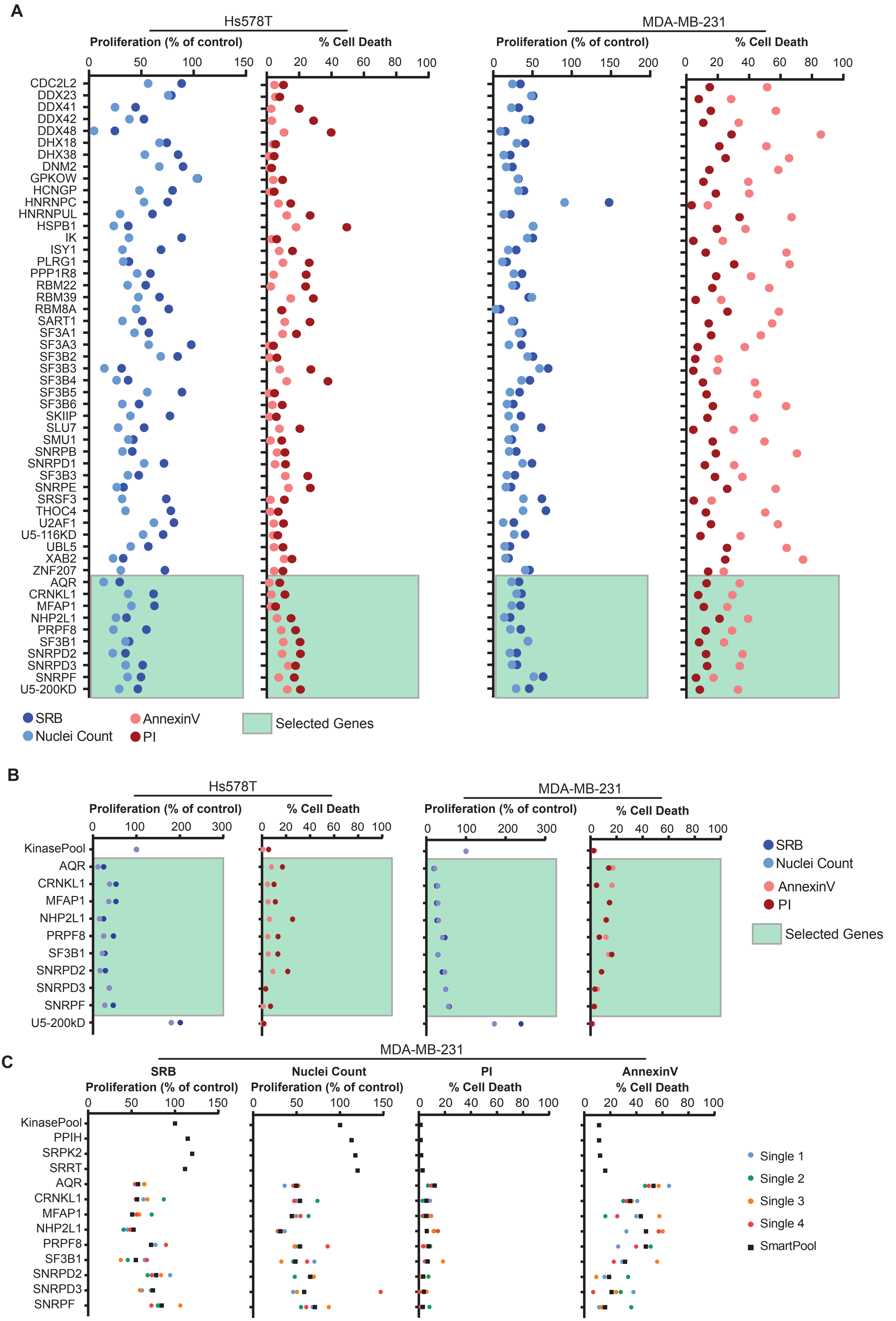

Supplemental Figure 2. ***Spliceosome RNAi validation screens.*** (A) Results of validation screen; effect of splicing factor knockdown on proliferation (SRB and nuclei count) and cell death (Annexin V and PI staining). Factors highlighted in green were selected for further validation. (B) Validation of candidate splicing factor SMARTpool knockdown on proliferation (SRB and Nuclei count) and cell death (PI and AnnexinV) in Hs578T (left) and MDA-MB-231 (right). Splicing factors selected for further validation are highlighted in green. (C) Validation of selected splicing factors using 4 single and SMARTpool siRNAs in MDA-MB-231. All measurements were performed 72 hours after knockdown.

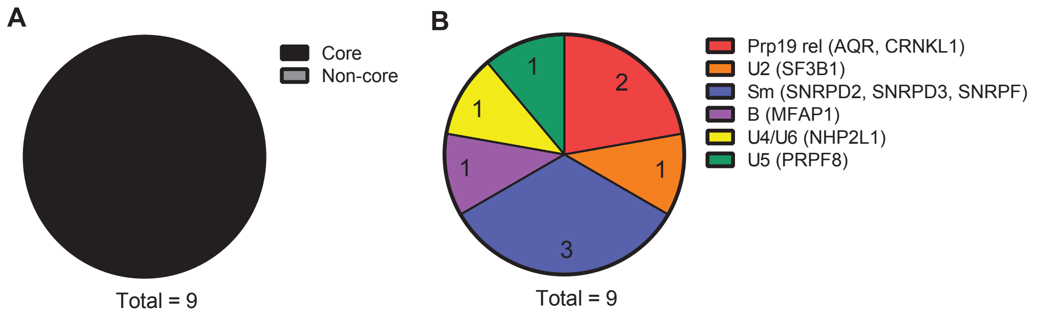

Supplemental Figure 3. ***Spliceosome candidate distribution over different subcomplexes.*** (A) Candidate distribution over core and non-core complexes. (B) Candidate distributation of functional splicing subcomplexes.

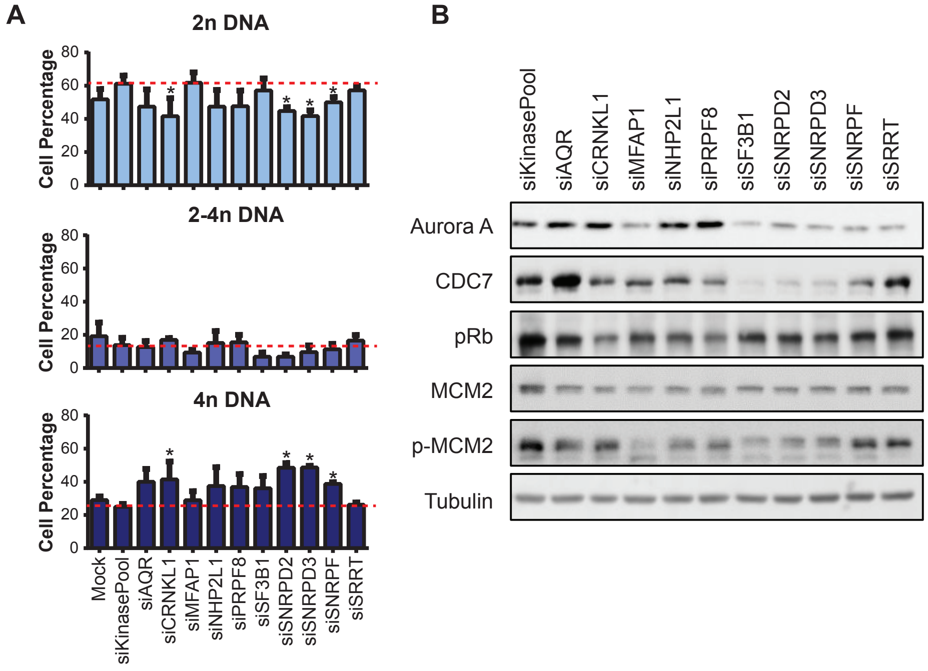

Supplemental Figure 4. ***Effect of candidate splicing factor knockdown on cell death and cell cycle progression in MDA-MB-231.*** (A) Effect of splicing factor knockdown on cell DNA content measured by FACS analysis in MDA-MB-231 cells 72 hours after transfection. Mean + stdev of three biological replicates. SRRT is a splicing factor not affecting proliferation and was used as negative control. (B) Effect of splicing factor knockdown on expression levels of cell cycle regulators in MDA-MB-231 cells 72 hours after transfection. Representative blots of two biological replicates. SRRT is a splicing factor not affecting proliferation and was used as negative control.Statistical significance was determined using ANOVA correcting for multiple testing. * $p < 0.05$, ** $p < 0.01$, *** $p < 0.001$.

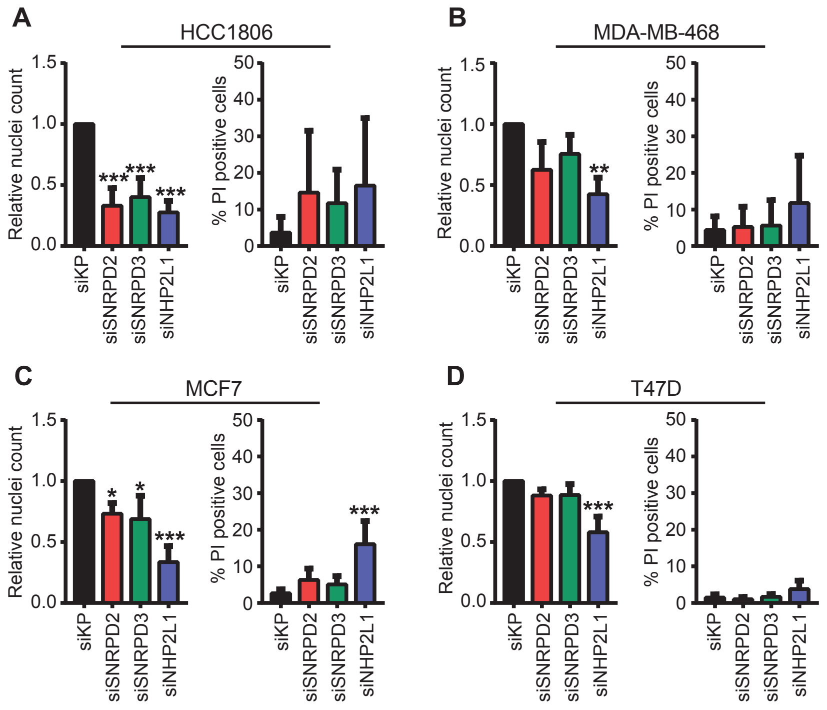

Supplemenatal Figure 5. *Effect of SNRPD2, SNRPD3 and NHP2L1 knockdown in other cell lines.* Effect of SNRPD2, SNRPD3 and NHP2L1 knockdown in proliferation and cell death three days after knockdown in HCC1806 (A), MDA-MB-468 (B), MCF7 (C) and T47D (D) cells. Mean + stdev of 3 biological replicates. Significance was determined using ANOVA correcting for multiple testing.

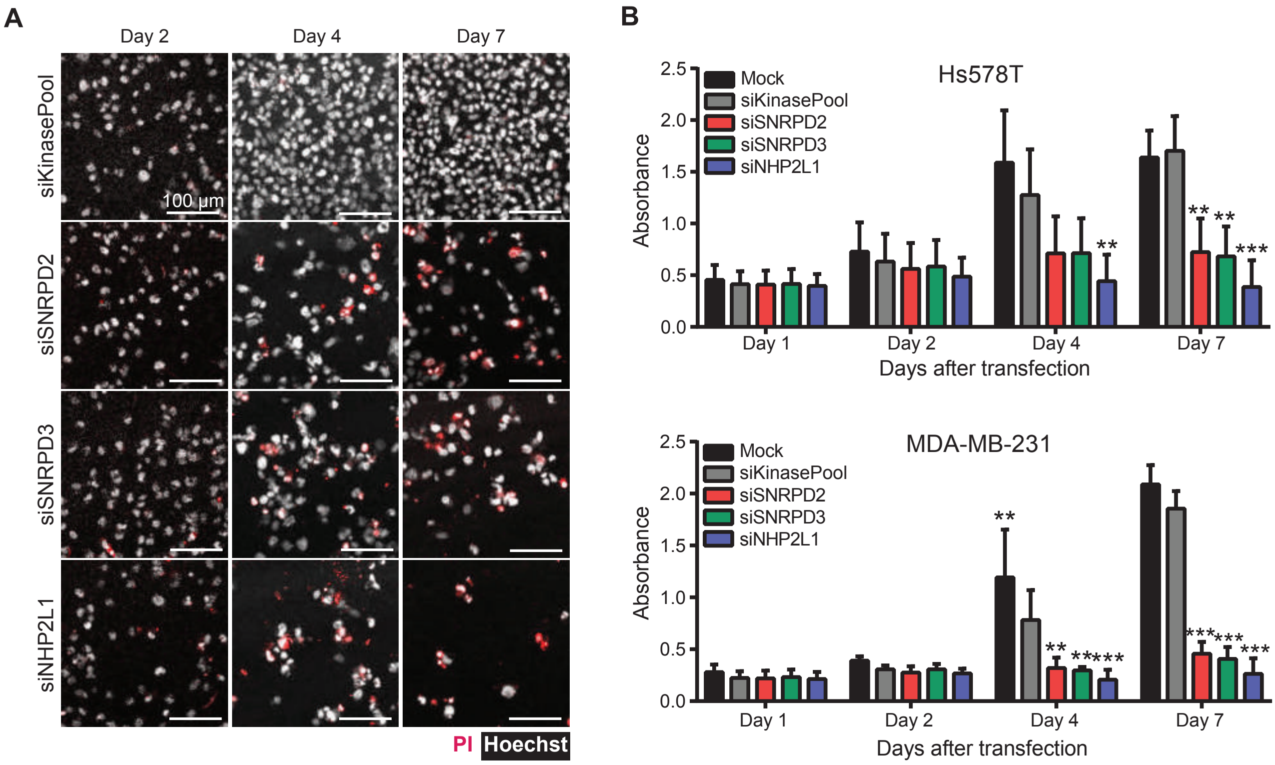

Supplemental Figure 6. ***SNRPD2, SNRPD3 and NHP2L1 knockdown reduce proliferation and induce cell death.*** (A) Representative images of propidium iodide (PI) and Hoechst staining 2, 4 and 7 days after knockdown in MDA-MB-231 cells. Scale bar = 100 µm. (B) Cell number 2, 4 and 7 days after splicing factor knockdown in Hs578T (top) and MDA-MB-231 (bottom) cell lines. Cell growth was measured using the SRB absorbance. Mean + stdev of three biological replicates. Statistical significance was calculated using ANOVA correcting for multiple testing. * $p < 0.05$, ** $p < 0.01$, *** $p < 0.001$.

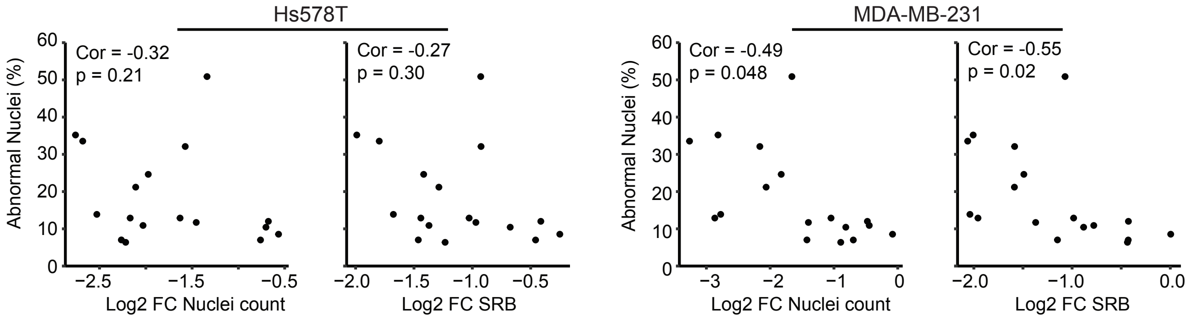

Supplemental Figure 7. ***Correlation primary screen with mitotic screen Sundamoorthy et al, 2014.***

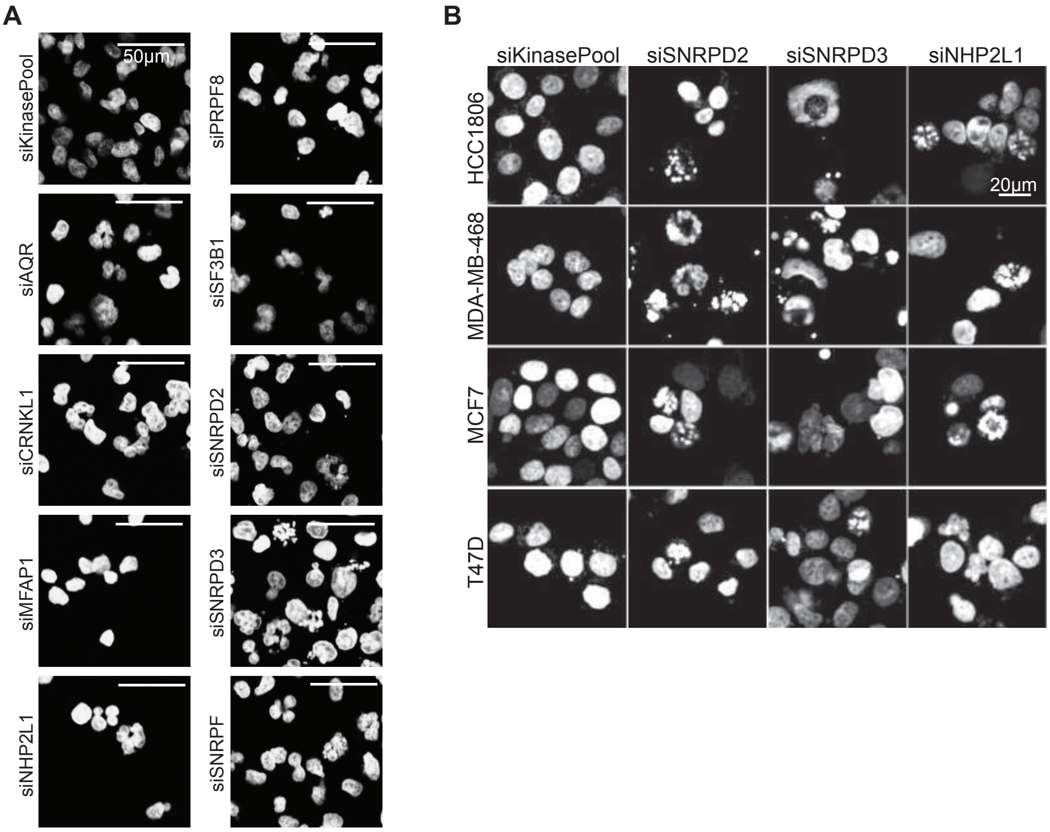

Supplemenatal Figure 8. ***Effect of SNRPD2, SNRPD3 and NHP2L1 on nuclear phenotype.*** (A) Nuclear phenotype 72 hours after splicing factor knockdown in MDA-MB-231 cells. Scale bar = 50 µm. (B) Effect of SNRPD2, SNRPD3 and NHP2L1 knockdown on nuclear phenotype in HCC1806, MDA-MB-468, MCF7 and T47D cells 72 hours after transfection. Scale bar = 20 µm.

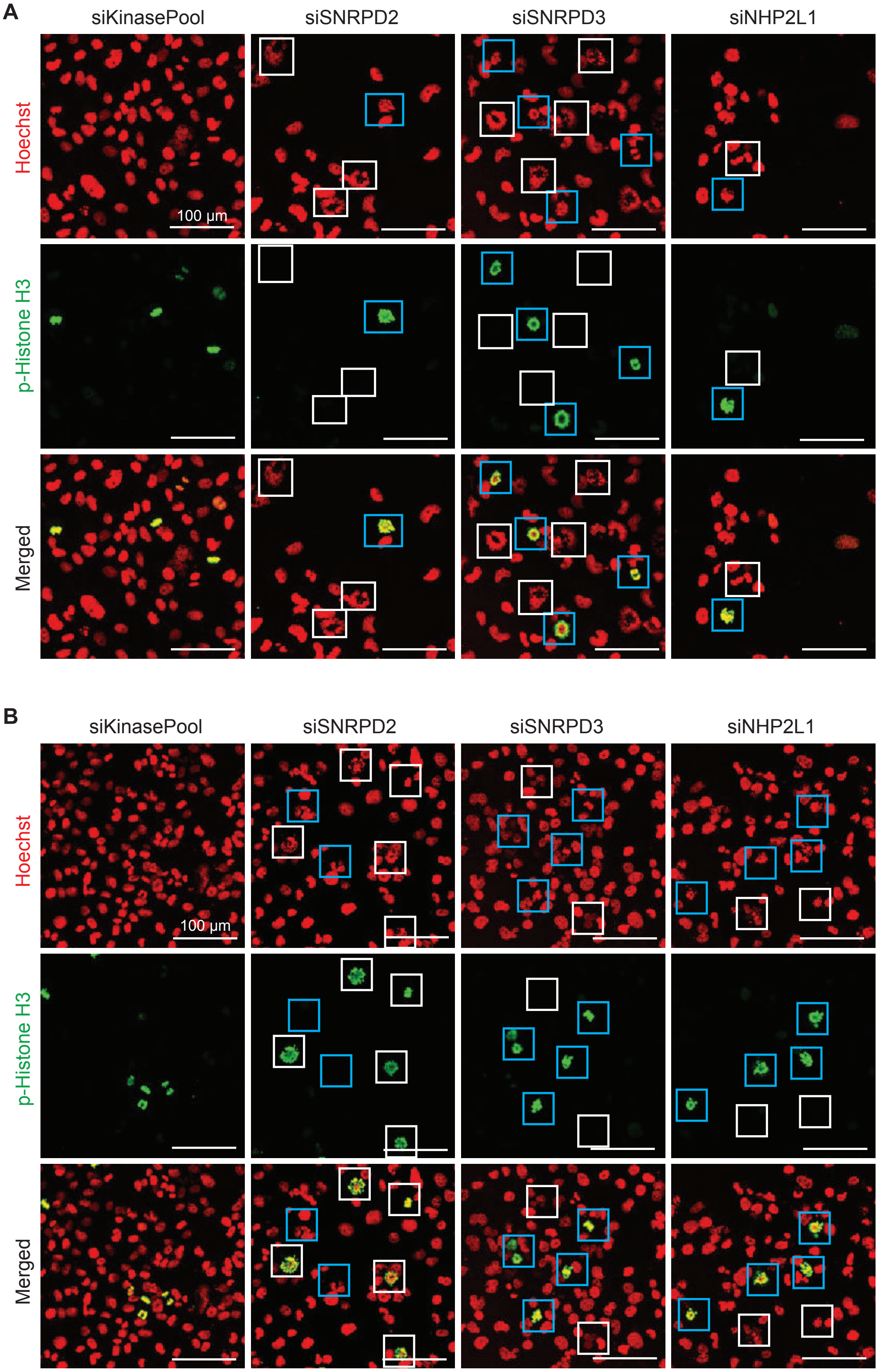

Supplemental Figure 9. ***Effect of SNRPD2, SNRPD3 and NHP2L1 knockdown on nuclear phenotype and mitosis.*** (A) Nuclear phenotype of p-histone H3 positive cells 72 hours after SNRPD2, SNRPD3 or NHP2L1 knockdown in Hs578T. Some cells with irregular nuclear phenotype are p-histone H3 positive (white squares), while some are negative (blue squares). (B) Similar as A, but for MDA-MB-231. Scale bar = 100µm.

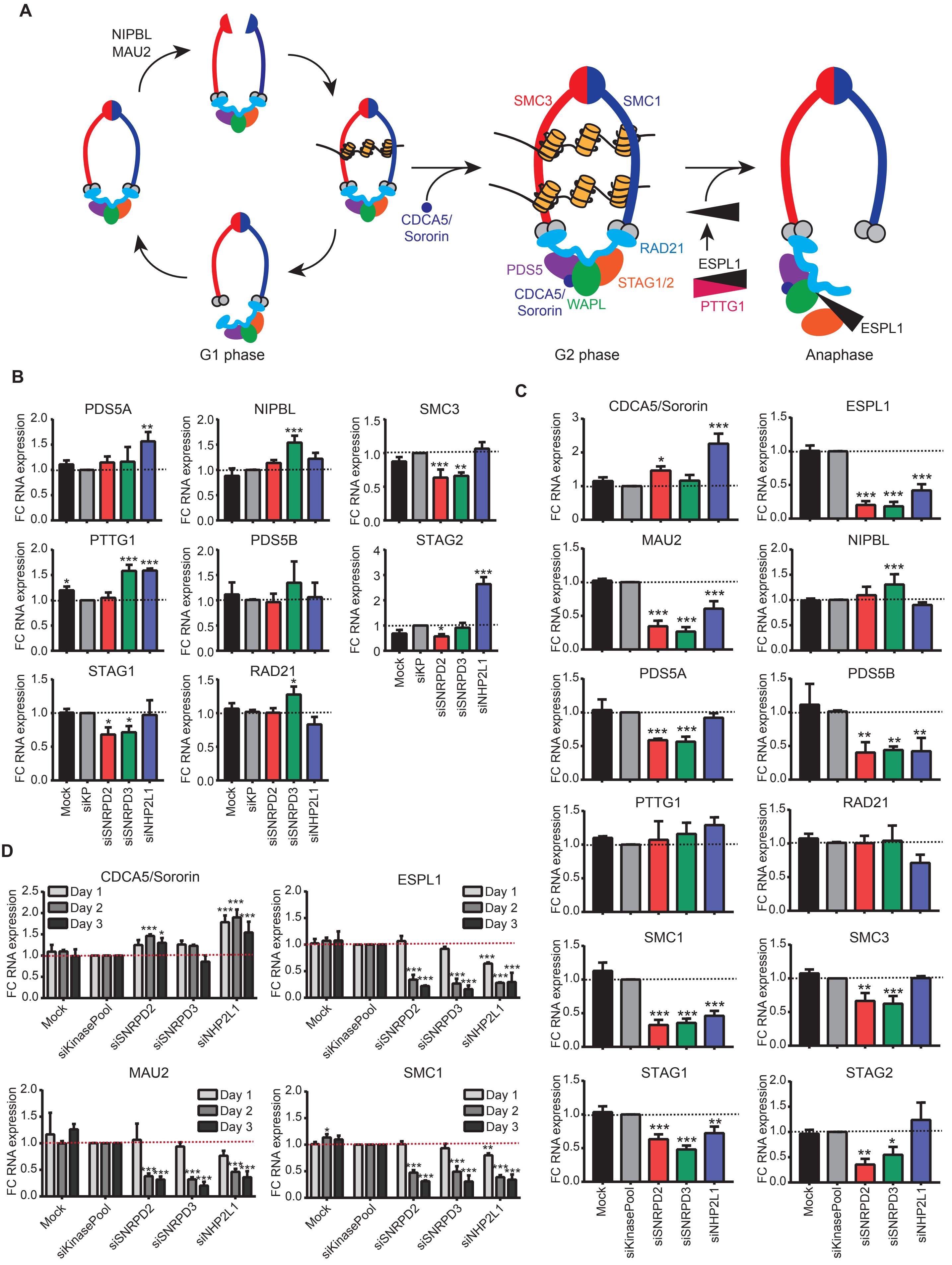

Supplemenatal Figure 10. ***Effect of candidate splicing factor knockdown on sister chromatid cohesion components.*** (A) Overview of the factors involved in sister chromatid cohesion. Adapted from Peters et al, 2012. (B) Effect of 72 hours SNRPD2, SNRPD3 and NHP2L1 knockdown on RNA expression levels of genes involved in sister chromatid cohesion in Hs578T. Mean + sd of 3 biological replicates.(C) Effect of 72 hours SNRPD2, SNRPD3 and NHP2L1 knockdown on RNA expression levels of genes involved in sister chromatid cohesion in MDA-MB-231. Mean + sd of 3 biological replicates. (D) Effect of SNRPD2, SNRPD3 and NHP2L1 knockdown on sororin, ESPL1, MAU2 and SMC1 expression levels 1, 2 and 3 days after knockdown in MDA-MB-231. Mean + sd of 3 biological replicates. Statistical significance was determined using ANOVA correcting for multiple testing. * $p < 0.05$, ** $p < 0.01$, *** $p < 0.001$.

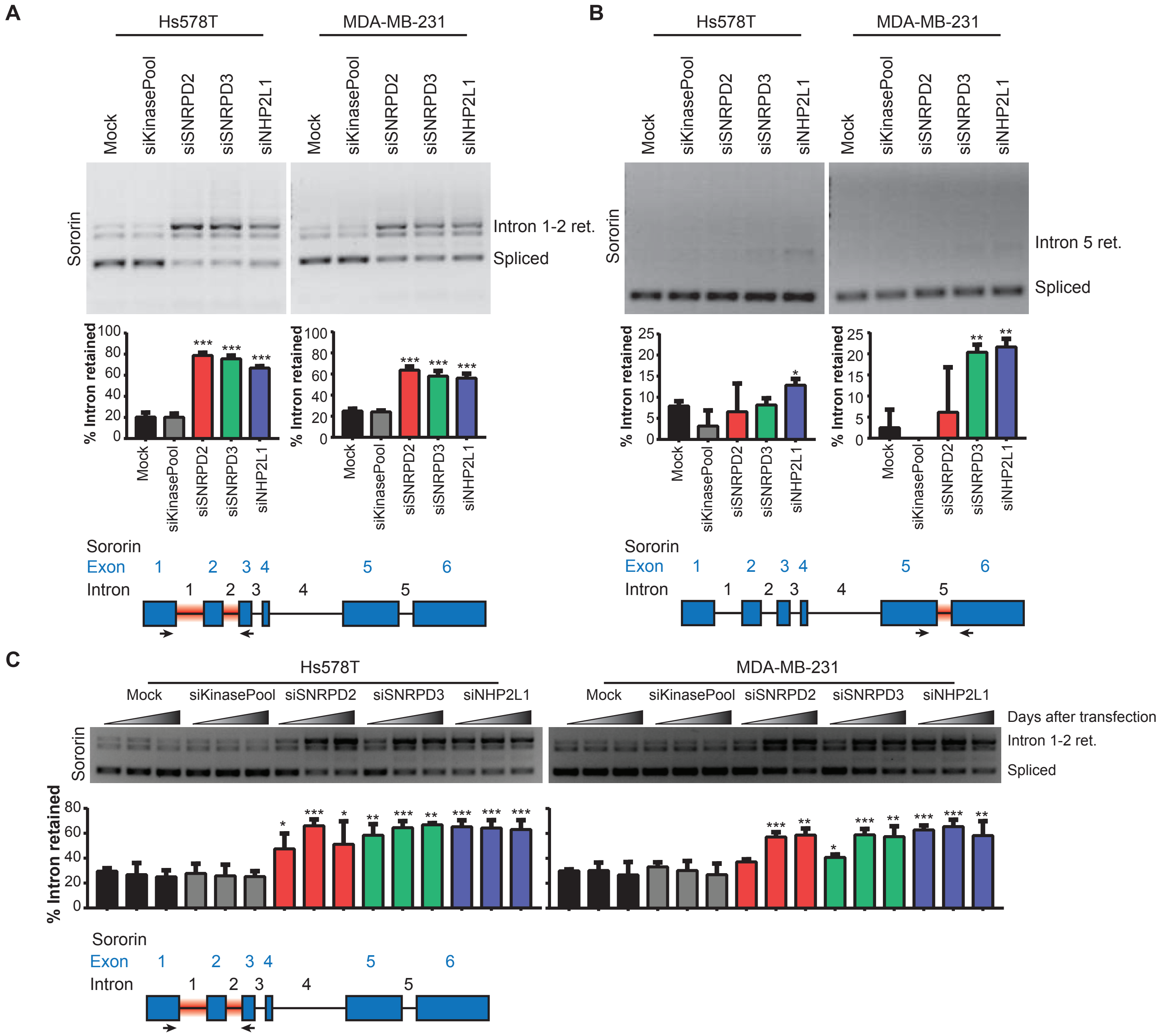

Supplemental Figure 11. ***Effect of SNRPD2, SNRPD3 and NHP2L1 knockdown on sororin intron retention.*** (A) Effect of 72 hours SNRPD2, SNRPD3 and NHP2L1 knockdown on sororin intron 1 and 2 retention in Hs578T and MDA-MB-231 cells. Mean + sd of three biological replicates. (B) Effect of 72 hours SNRPD2, SRNPD3 and NHP2L1 knockdown on sororin intron 5 retention in Hs578T and MDA-MB-231 cells. Mean + sd of three biological replicates. (C) Effect of 72 hours SNRPD2, SNRPD3 and NHP2L1 knockdown on sororin intron 1 and 2 retention 1, 2 and 3 days after knockdown in Hs578T and MDA-MB-231. Mean + sd of three biological replicates. Statistical significance was determined using ANOVA correcting for multiple testing. * $p < 0.05$, ** $p < 0.01$, *** $p < 0.001$.

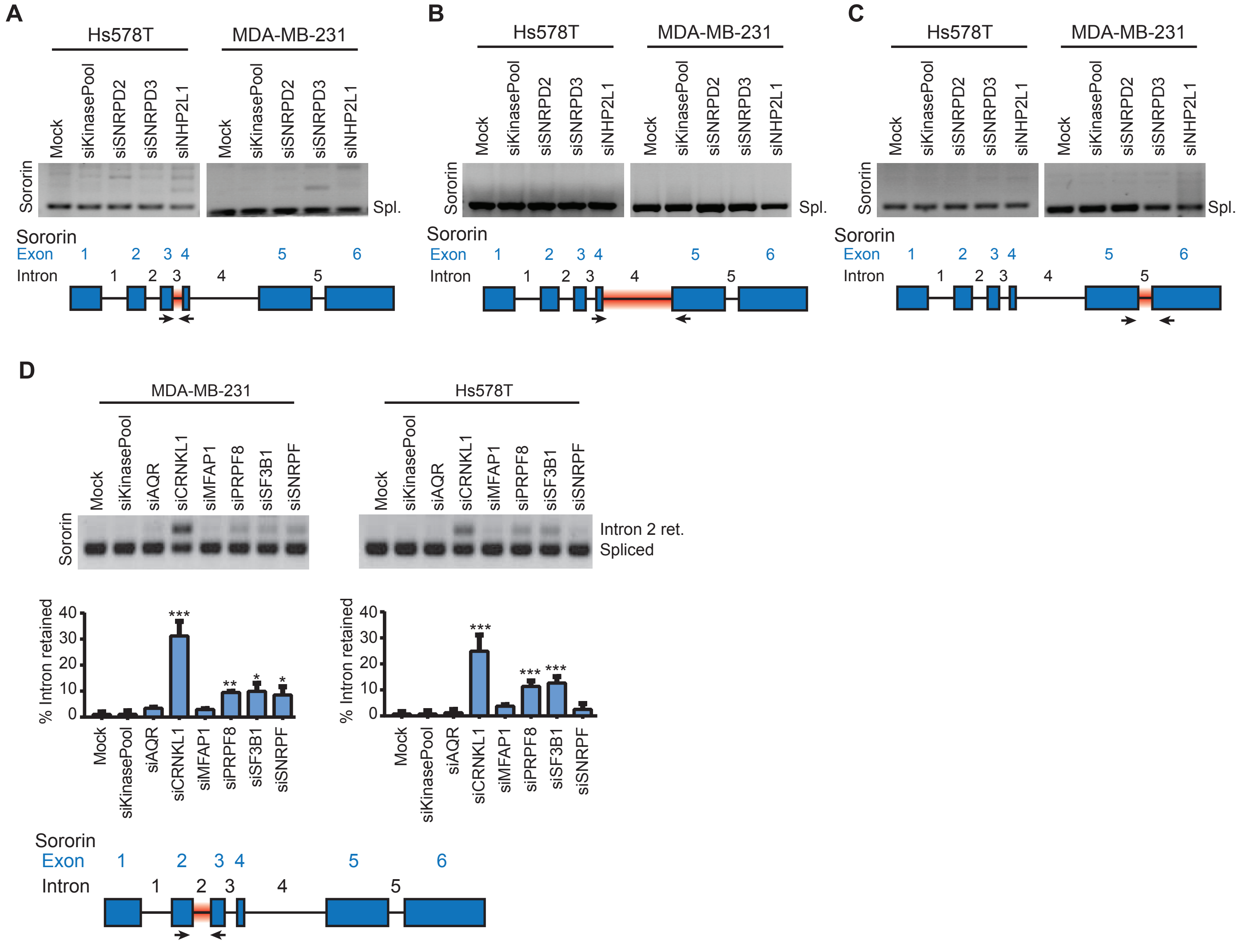

Supplemental Figure 12. ***Effect of candidate splicing factor knockdown on sororin intron retention.*** Effect of SNRPD2, SNRPD3 and NHP2L1 knockdown on sororin intron 3 (A), intron 4 (B) or intron 5 (C) retention in Hs578T and MDA-MB-231 cell lines 72 hours after transfection. (D) Effect of candidate splicing factor knockdown on sororin intron 2 retention in MDA-MB-231 and Hs578T cells 72 hours after transfection. SRRT is a splicing factor not affecting TNBC proliferation and was used as a negative control. Mean + sd of three biological replicates. Mean + sd of three biological replicates. Statistical significance was determined using ANOVA correcting for multiple testing. * $p < 0.05$, ** $p < 0.01$, *** $p < 0.001$.

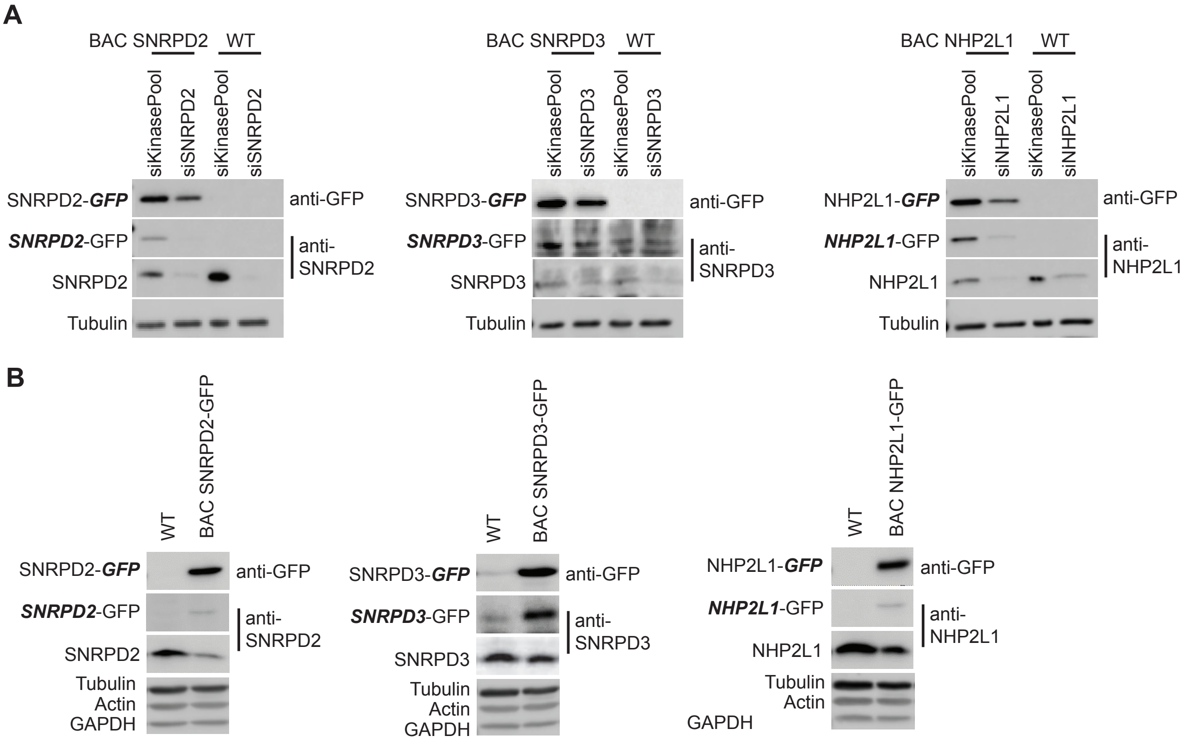

Supplemental Figure 13. ***SNRPD2, SNRPD3 and NHP2L1 Hs578T BAC-GFP validation.*** (A) Endogenous and GFP fusion protein levels of SNRPD2, SNRPD3 and NHP2L1 in Hs578T BAC reporter cell lines 72 hours after control and target knockdown. (B) Endogenous and GFP fusion protein levels in Hs578T wild type and BAC reporter cell lines.

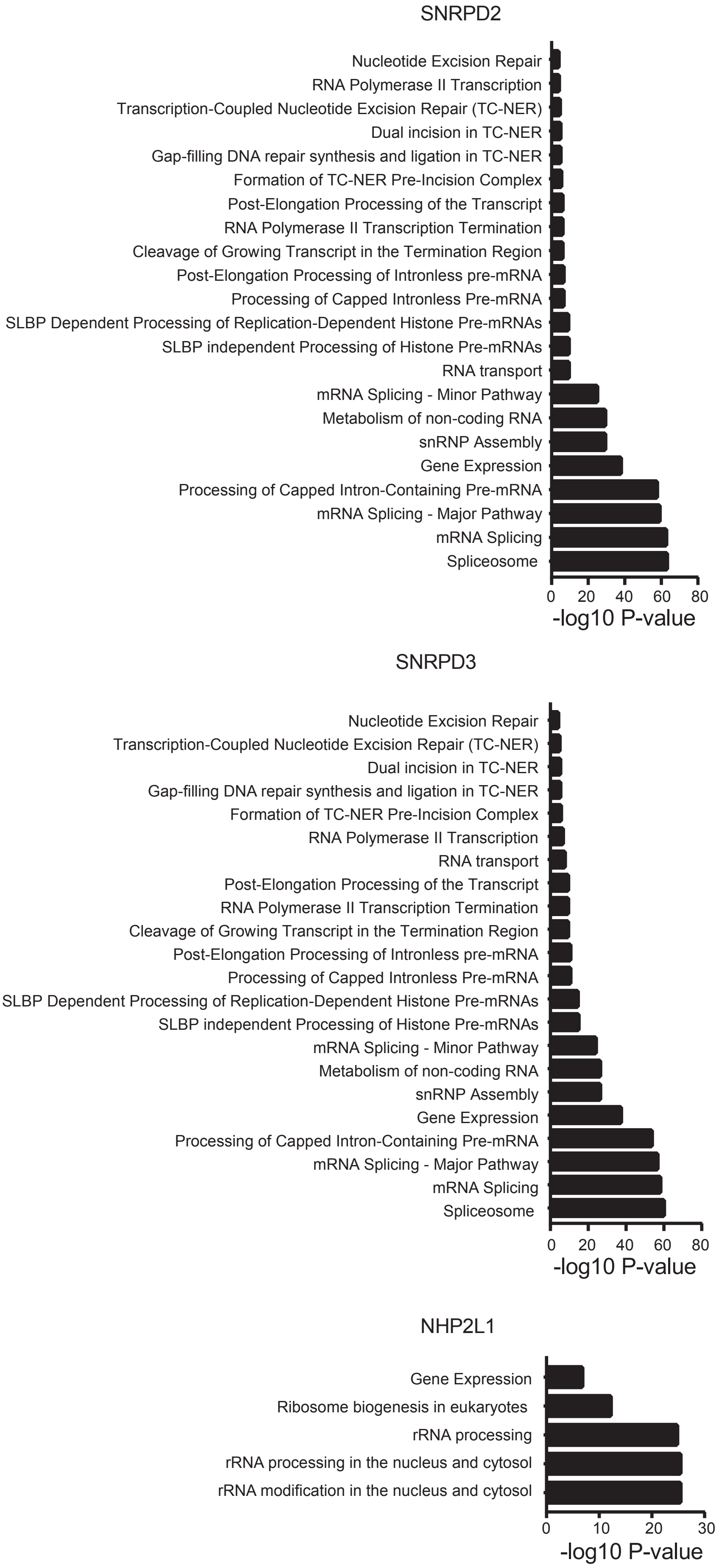

Supplemental Figure 14. ***Overrepresentation analysis of proteins in complex with SNRPD2, SNRPD3 or NHP2L1.*** Significantly enriched proteins were used for overrepresentation analysis using ConsensusPathDB (Kamburov) for Reactome and KEGG pathways.

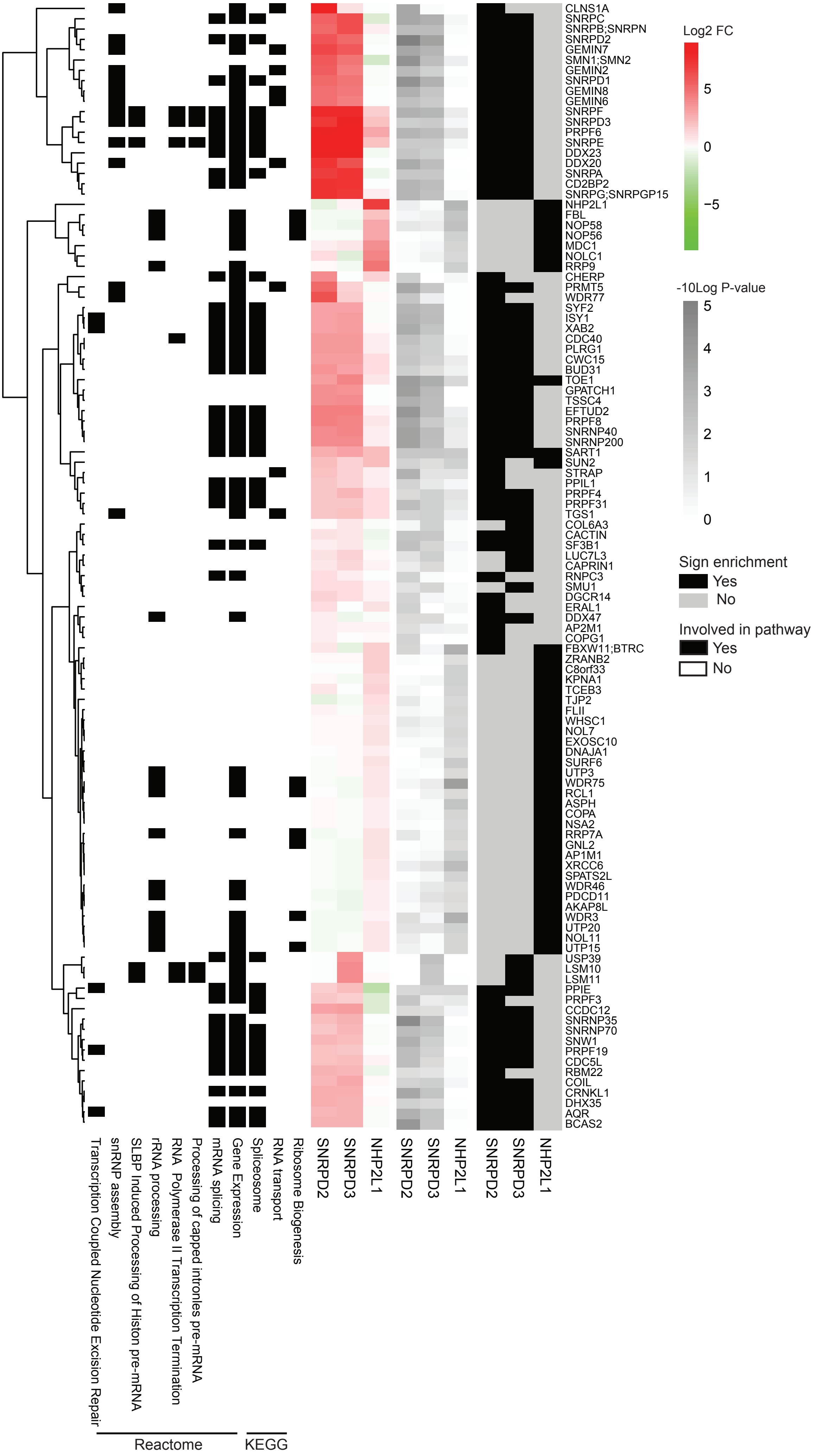

Supplemental Figure 15. ***Hierarchical clustering of proteins enriched in at least one of the splicing factor (SNRPD2, SNRPD3 or NHP2L1) complexes.*** From right to left; 1) involvement in commonly overrepresented pathways, 2) log2 fold change enrichment in splicing factor complex, 3) -log10 adjusted P-value for enrichment in splicing factor complex and 4) significance of the protein associated with the SNRPD2, SNRPD3 or NHP2L1 complex.

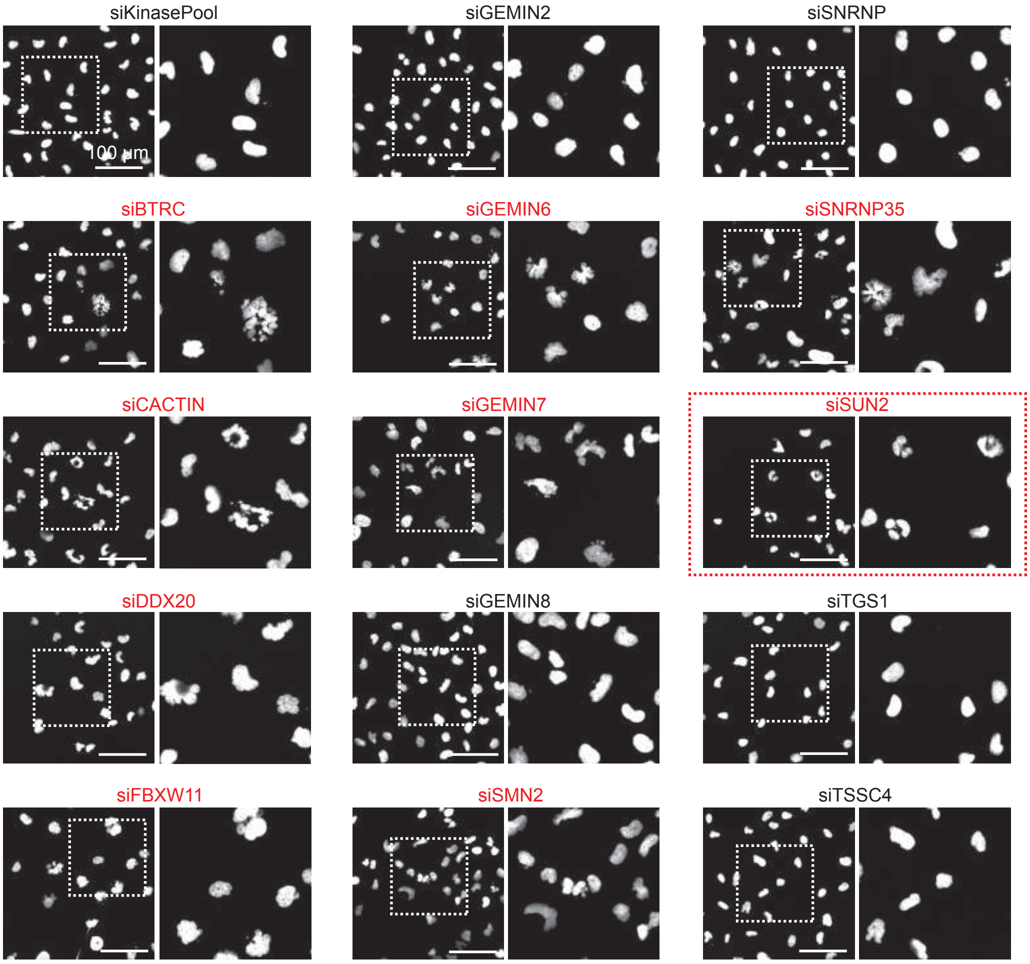

Supplemental Figure 16. ***Nuclear phenotype upon knockdown of components in splicing factor complexes.*** Hoechst staining 72 hours after transfection in Hs578T cells. Red labeled factors show abnormal nucleur phenotype upon knockdown. Scale bar = 100 µm. Right: zoom in of dashed square in left image.

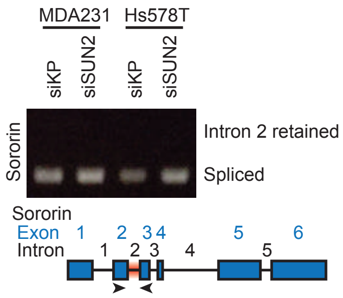

Supplemental Figure 17. ***Sororin intron 2 retention after SUN2 knockdown in MDA-MB-231 and Hs578T cell lines.***
